# Leaf volatile and nonvolatile metabolites show different levels of specificity in response to herbivory

**DOI:** 10.1002/ece3.10123

**Published:** 2023-05-29

**Authors:** Priscila Mezzomo, Alexander Weinhold, Klára Aurová, Leonardo R. Jorge, Petr Kozel, Jan Michálek, Nela Nováková, Carlo L. Seifert, Tereza Volfová, Marica Engström, Juha‐Pekka Salminen, Brian E. Sedio, Martin Volf

**Affiliations:** ^1^ Biology Centre CAS Institute of Entomology Ceske Budejovice Czech Republic; ^2^ Faculty of Science University of South Bohemia Ceske Budejovice Czech Republic; ^3^ German Centre for Integrative Biodiversity Research (iDiv) Halle‐Jena‐Leipzig Leipzig Germany; ^4^ Institute of Biodiversity University of Jena Jena Germany; ^5^ Centre Algatech CAS Institute of Microbiology Třeboň Czech Republic; ^6^ Biology Centre CAS Institute of Parasitology Ceske Budejovice Czech Republic; ^7^ Department of Forest Nature Conservation, Faculty of Forest Sciences and Forest Ecology Georg‐August‐University Göttingen Göttingen Germany; ^8^ Department of Chemistry University of Turku Turku Finland; ^9^ Department of Integrative Biology University of Texas at Austin Austin Texas USA; ^10^ Smithsonian Tropical Research Institute Balboa, Ancón Republic of Panama

**Keywords:** chemical diversity, defense specificity, herbivory, induction, *Salix*, specialized metabolites, volatile organic compounds

## Abstract

Plants produce diverse chemical defenses with contrasting effects on different insect herbivores. Deploying herbivore‐specific responses can help plants increase their defensive efficiency. Here, we explore how variation in induced plant responses correlates with herbivore species, order, feeding guild, and level of specialization. In a greenhouse experiment, we exposed 149 plants of *Salix fragilis* (Linnaeus, 1753) to 22 herbivore species naturally associated with this host. The insects belonged to four orders (Coleoptera, Lepidoptera, Hemiptera, and Hymenoptera), three feeding guilds (external leaf‐chewers, leaf‐tying chewers, and sap‐sucking), and included both dietary specialists and generalists. Following herbivory, we quantified induced changes in volatiles and nonvolatile leaf metabolites. We performed multivariate analyses to assess the correlation between herbivore order, feeding guild, dietary specialization, chewing damage by herbivores, and induced responses. The volatile composition was best explained by chewing damage and insect order, with Coleoptera and Lepidoptera eliciting significantly different responses. Furthermore, we recorded significant differences in elicited volatiles among some species within the two orders. Variation in nonvolatile leaf metabolites was mainly explained by the presence of insects, as plants exposed to herbivores showed significantly different metabolites from controls. Herbivore order also played a role to some extent, with beetles eliciting different responses than other herbivores. The induction of volatile and nonvolatile leaf metabolites shows different levels of specificity. The specificity in volatiles could potentially serve as an important cue to specialized predators or parasitoids, increasing the efficacy of volatiles as indirect defenses. By contrast, the induction of nonvolatile leaf metabolites was largely unaffected by herbivore identity. Most nonvolatile metabolites were downregulated, possibly indicating that plants redirected their resources from leaves in response to herbivory. Our results demonstrate how diverse responses to herbivores can contribute to the diversity of plant defensive strategies.

## Graphical abstract

We test how insect herbivore species, order, feeding guild and level of specialization correlate with the variation in induced plant responses. The induction of volatile and nonvolatile leaf metabolites shows different levels of specificity. Our results demonstrate how diverse responses to herbivores can contribute to the diversity of defensive plant strategies.

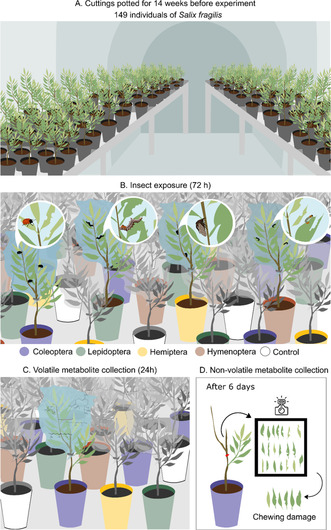

## 1 INTRODUCTION

Plants have evolved many defensive strategies to cope with insect herbivores (Janz, 2011). The proliferation of plant defenses has supported the evolution of diverse feeding strategies and counter‐adaptations in insects (Stam et al., 2014). As a result, different insect herbivores often show differential responses to plant defenses—a defense acting against one herbivore can fail to affect another (Herrera & Pellmyr, 2002). Therefore, deploying herbivore‐specific responses can increase plant defense efficiency (Erb et al., 2012). Evidence shows that tailoring macroevolutionary defensive trajectories can help plants escape herbivory on the evolutionary scale (Volf et al., 2018). However, specificity in plant defenses also acts on finer temporal scales where tailored induced responses can rapidly improve their efficacy (Mattiacci et al., 2001).

In response to herbivory, plants can employ various induced defenses that target insect herbivores directly, such as mechanical and chemical defensive traits (Herrera & Pellmyr, 2002). Direct chemical defenses include diverse specialized metabolites that impact herbivores' performance, host preference, fecundity, or survival (Chen, 2008). While generalist herbivores are typically negatively affected by direct defenses, some specialized herbivores have evolved mechanisms to overcome their impact (Ali & Agrawal, 2012). Plants with herbivore damage can also upregulate the production of various volatile organic compounds (VOCs) that attract natural enemies of herbivores (Sobhy et al., 2017). The production of some VOCs increases immediately after herbivory, while others can show a slower emission or only increase on the next photoperiod (i.e., day) after herbivory (Mattiacci et al., 2001). Nevertheless, many VOCs are readily deployable, unlike nonvolatile leaf metabolites that may take days or weeks to be synthesized or relocated in response to herbivory (Boeckler et al., 2011; Chen, 2008).

Induced responses in plant chemistry can markedly differ upon herbivory by insects from different feeding guilds or with different levels of dietary specialization (Ali & Agrawal, 2012). For example, the wound inflicted by sap‐sucking vs. leaf‐chewer herbivores induces largely differential responses in the host (Danner et al., 2018; Dicke et al., 2009; Stam et al., 2014). Whereas leaf‐chewers usually damage large amounts of tissue, sap‐suckers cause a smaller wound while feeding on the phloem, xylem, or mesophyll cells. Another layer of specificity arises from the presence of specific chemical elicitors in their oral secretions (Sobhy et al., 2017). Related herbivores likely show some level of conservatism in their oral secretions, which can support similar induced responses (Shinya et al., 2016). The presence of specialized salivary enzymes can be a key component in regulating induced plant defenses by specialist herbivores, which could further partition responses among related generalists and specialist insects (Sobhy et al., 2017). Such specificity in VOC signals may promote efficiency in communication among plants, herbivores, and their natural enemies since they carry information on herbivore identity and suitability as prey (Clavijo McCormick et al., 2012; Danner et al., 2018; Mumm & Dicke, 2010).

Deploying specific responses to different herbivores may be especially important in perennial woody plants that harbor herbivores from various taxa and feeding guilds (Danner et al., 2018). These plants encounter a broad spectrum of insects during their lifetimes, and they are integrated into complex multitrophic webs where interaction specificity often plays a key role (Clavijo McCormick et al., 2014). In this sense, deploying localized or specific induced responses can improve their efficacy (Volf et al., 2021). Indeed, trees infested by various herbivore orders can elicit differential emissions of terpenes and nitrogenous compounds, groups of VOCs that play important roles in the attraction of natural enemies (Clavijo McCormick et al., 2014).

Given the diversity of herbivores that woody plants typically harbor, they allow testing the specificity in induced responses in plant‐herbivore interactions. Measuring the specificity in induced plant defenses using naturally associated plants and herbivores is much needed to explore these trends. In this regard, willows (genus *Salix*) represent an excellent and ecologically relevant study system. Willows harbor diverse insect communities, serving as a keystone plant genus for supporting herbivore diversity (Narango et al., 2020). Willows possess diverse defensive chemistry, which drives differential insect responses (Volf, Hrcek, et al., 2015). Nonvolatile defenses of willows are primarily phenolic‐based. In addition to various tannins and flavonoids, they include salicinoids, a group of phenolic glycosides characteristic of the Salicaceae family (Boeckler et al., 2011; Volf, Hrcek, et al., 2015; Volf, Julkunen‐Tiitto, et al., 2015). Salicinoids affect generalist herbivores by acting as feeding deterrents, retarding larval growth, or increasing mortality (Kolehmainen et al., 1995). By contrast, several specialized herbivores have adapted to salicinoids and use them as feeding or oviposition cues. Some *Chrysomela* or *Phratora* leaf beetle larvae use salicinoids as substrates for producing anti‐predator defenses (Pasteels et al., 1983; Rank et al., 1998). Willows also produce various monoterpenes that can attract predators (Mrazova & Sam, 2018). Many specialized leaf beetles induce strong responses in VOCs from poplars and willows (Peacock et al., 2001; Unsicker et al., 2015). Unlike nonvolatile defenses that may be less efficient against specialists, VOCs could thus be an alternative defense strategy by attracting predators and parasitoids (Ali & Agrawal, 2012).

Here, we explore how variation in induced plant responses correlates with herbivore species, order, feeding guild, and level of specialization. We build on previous work by Danner et al. (2018) who analyzed how herbivore diet breadth and feeding guild affect VOC profiles in *Brassica rapa*. We expand the experiments into a diverse plant‐insect system and compare the specificity between VOCs and nonvolatile leaf metabolites. We used 22 species of herbivores naturally associated with the crack willow (*Salix fragilis* L.) and show the relative importance of herbivore feeding guild, order, specialization, and species on specificity in induced plant responses. We formulated three expectations: (i) There will be higher specificity in VOCs than in nonvolatile leaf metabolites as their efficacy in communication among plants, herbivores, and their natural enemies probably largely depend on their specificity. (ii) Most of the induced responses will be correlated to the herbivore order or feeding guild since herbivores from the same orders or guilds are expected to show similar responses. (iii) Herbivores with different levels of dietary specialization will trigger differential responses; generalist herbivores will elicit responses in both VOCs and nonvolatile leaf metabolites, and specialized herbivores will primarily elicit responses in VOCs that can attract specialized predators and parasitoids.

## 2 MATERIALS AND METHODS

### 2.1 Host plants

We studied *Salix fragilis*, commonly known as the crack willow, a dominant willow species that harbor rich assemblages of insect herbivores. We obtained a total of 209 cuttings from two individuals of *S. fragilis* (111 cuttings from individual A and 98 cuttings from individual B) near Ceske Budejovice, Czech Republic (49.0123 N, 14.4939 E) on February 4th, 2020. Using clonal cuttings from only two plants allowed us to minimize intraspecific variation among the plants and detect the variation in induced responses due to specificity in feeding by various herbivores. Detailed information on plant treatment prior to the experiment is available in Appendix S1.

### 2.2 Model insects

We exposed the willow cuttings to 22 species of insect herbivores (Table 1). Before the experiment, 19 of the herbivore species were obtained as adults or larvae from field collections near Ceske Budejovice (CZ). The remaining three species were obtained from overwintered eggs of long‐term colonies (*Lymantria dispar*) or temporary rearings (*Operophtera brumata* and *Catocala nupta*). The insects belonged to four orders (Coleoptera, Lepidoptera, Hemiptera, and Hymenoptera) and three feeding guilds: external leaf‐chewers (including Coleoptera, Lepidoptera, and Hymenoptera), leaf‐tying chewers (Lepidoptera), and sap‐suckers (Hemiptera). We decided to divide leaf‐chewing herbivores into external leaf‐chewers and leaf‐tying chewers as these groups can have different natural enemies (Tvardikova & Novotny, 2012) and affect the plants in different ways since leaf‐tying chewers alter leaf photosynthetic activity or can induce premature senescence (Lind et al., 2001; Nabity et al., 2009).

**TABLE 1:** List of herbivore species, their order, feeding guild, dietary specialization, number of replicates per insect species, and the number of individuals added to each plant (in brackets).

Herbivore	Life stage	Order	Feeding guild	Diet breadth	*N*
*Chrysomela populi* (Linnaeus, 1758)	Adult	Coleoptera	Leaf‐chewer	Specialist	8 [2]
*Chrysomela populi*	Larvae	Coleoptera	Leaf‐chewer	Specialist	8 [4]
*Chrysomela cuprea* (Fabricius, 1775)	Adult	Coleoptera	Leaf‐chewer	Specialist	8 [3]
*Chrysomela cuprea*	Larvae	Coleoptera	Leaf‐chewer	Specialist	8 [6]
*Gonioctena decemnotata* (Marsham, 1802)	Larvae	Coleoptera	Leaf‐chewer	Specialist	8 [8]
*Phyllobius pyri* (Linnaeus, 1758)	Adult	Coleoptera	Leaf‐chewer	Generalist	6 [4]
*Phyllobius viridicollis* (Fabricius, 1792)	Adult	Coleoptera	Leaf‐chewer	Generalist	8 [6]
*Plagiodera versicolora* (Laicharting, 1781)	Adult	Coleoptera	Leaf‐chewer	Specialist	8 [6]
*Aphropohora salicina* (Goeze, 1778)	Nymph	Hemiptera	Sap‐sucker	Specialist	8 [10]
*Pterocomma beulahense* (Cockerell, 1904)	Nymph + adult	Hemiptera	Sap‐sucker	Specialist	6 [100]
*Centrotus cornutus* (Linnaeus, 1758)	Adult	Hemiptera	Sap‐sucker	Generalist	4 [2]
*Amauronematus viduatus* (Zetterstedt, 1838)	Larvae	Hymenoptera	Leaf‐chewer	Specialist	8 [2]
*Amauronematus* sp.	Larvae	Hymenoptera	Leaf‐chewer	Specialist	6 [2]
*Lymantria dispar* (Linnaeus, 1758)	Larvae	Lepidoptera	Leaf‐chewer	Generalist	6 [2]
*Operophtera brumata* (Linnaeus, 1758)	Larvae	Lepidoptera	Leaf‐tying chewer	Generalist	4 [2]
*Erannis defoliaria* (Clerck, 1759)	Larvae	Lepidoptera	Leaf‐chewer	Generalist	8 [1]
*Orgyia antiqua* (Linnaeus, 1758)	Larvae	Lepidoptera	Leaf‐chewer	Generalist	6 [1]
*Catocala nupta* (Linnaeus, 1767)	Larvae	Lepidoptera	Leaf‐chewer	Specialist	6 [1]
*Agriopis marginaria* (Fabricius, 1776)	Larvae	Lepidoptera	Leaf‐chewer	Generalist	6 [1]
*Agrochola lota* (Clerck, 1759)	Larvae	Lepidoptera	Leaf‐tying chewer	Generalist	7 [1]
*Cosmia trapezina* (Linnaeus, 1758)	Larvae	Lepidoptera	Leaf‐chewer	Generalist	7 [1]
*Anacampsis populella* (Clerck, 1759)	Larvae	Lepidoptera	Leaf‐tying chewer	Generalist	6 [2]
*Agonopterix conterminella* (Zeller, 1839)	Larvae	Lepidoptera	Leaf‐tying chewer	Specialist	4 [1]
*Amphipyra pyramidea* (Linnaeus, 1758)	Larvae	Lepidoptera	Leaf‐chewer	Generalist	4 [1]

We further classified the insects as specialists or generalists depending on whether their host ranges include only members of the Salicaceae family or also other plants lineages, broadly following specialization categories proposed in other studies (Ali & Agrawal, 2012; Volf, Hrcek, et al., 2015). We extracted the information on host spectra from online databases, our previous studies, and the literature.

In our experiments, we used a combination of the last two larval instars for caterpillars and sawflies, the last two larval instars and adults for beetles, and nymphs and adults for hemipteran insects (Table 1). These life stages were selected to optimize insect handling and ensure sufficient damage to the experimental plants. We introduced a different number of individuals to the experimental plants to balance the expected damage across the insect species (Table 1). Since various leaf‐chewing species still consumed different amounts of leaf tissue during the experiment, we also included the chewing damage (i.e., leaf area loss) from all experimental plants in our analyses to account for this. We observed feeding by all sap‐sucking herbivores, such as the notable foam formation upon feeding of *Aphrophora salicina* individuals. Unfortunately, it was not possible to quantify the damage they caused to the plants since the damaged area was not visible as in the case of leaf‐tying chewer and external leaf‐chewer herbivores. To account for this, we originally included the weight of the sap‐sucking insects introduced to each plant in our preliminary analyses. As it did not have any effect on the results, we ultimately excluded the weight of sap‐suckers from the final models.

### 2.3 Experimental setup and sampling

We started the experiment on May 12th, 2020. We used a total of 149 cuttings of *S. fragilis*, out of which 139 plants were exposed to the herbivores, while 10 plants were selected as controls. We introduced the insects to the terminal part of the largest shoot on the plants with ca. 10 leaves and enclosed in 26 × 35 cm tissue bags. Control plants received bags without herbivores. We randomly distributed the plants in the greenhouse. We allowed insects to feed on the plants for 72 h and we checked them several times a day. We immediately replaced inactive or dead insects with conspecific individuals of the same developmental stage.

On May 15th, we removed all herbivores and their frass from the plants. We attached two PDMS (polydimethylsiloxane) tubes (2 cm cuttings, inner diameter 1.0 mm, outer diameter 1.8 mm, Carl Roth, Karlsruhe, Germany) to each plant immediately after removing the herbivores. PDMS tubes were placed on clean stainless steel wire to avoid contact with the plant surface and enclosed in 25 × 38 cm polyamide bags (Alufix Bohemia, Cerniky, Czech Republic). We passively sampled the VOCs from headspace for 24 h following Kallenbach et al. (2014). This method is particularly suitable for sampling monoterpenoids and sesquiterpenoids, two groups of VOCs we were interested in due to their roles in plant‐herbivore interactions in willows and poplars (Peacock et al., 2001).

In our previous studies, we typically encountered slow upregulation of nonvolatiles, following induction in various woody plant species (e.g., Thaler et al., 1996, 2001; Volf et al., 2021). We thus allowed six days after the VOCs sampling for the response in nonvolatile leaf metabolites to occur. Then, we collected and individually photographed all the leaves enclosed in the bags on all the treatment and control plants. We processed the images in ImageJ (Abramoff et al., 2004) to measure the leaf area and amount of chewing damage caused by the external leaf‐chewer and leaf‐tying chewer herbivores. To quantify leaf damage, we measured the total area of each leaf per individual plant and the area that was eaten by the herbivore. In the case of leaf‐tying chewers, we performed the measurements by unfolding the leaves tied by the caterpillars and flattening them with a sheet of glass, so the damage would clearly be visible. After measuring the chewing damage, we took the first three fully developed upper leaves from each plant, freeze‐dried them, and homogenized them for further chemical analyses.

### 2.4 Chemical analyses

#### 2.4.1 VOCs quantification

We analyzed the PDMS tubes by thermal desorption‐gas chromatography–mass spectrometry (TD‐GC–MS) in a thermodesorption unit (MARKES, Unity 2, Llantrisant, United Kingdom) equipped with an autosampler (MARKES, Ultra 50/50). TD‐GC–MS used the following conditions: carrier gas Helium (constant flow rate of 1 mL/min), flow path temperature 150°C; processing method: dry purge 5 min at 20 mL/min, prepurge 2 min at 20 mL/min, desorption 8 min at 280°C with 20 mL/min, pretrap fire purge 1 min at 30 mL/min, trap heated to 300°C and hold for 4 min. VOCs were separated on a gas chromatograph (Bruker, GC‐456, Bremen, Germany) connected to a triple‐quad mass spectrometer (Bruker, SCION) equipped with a DB‐WAX column (30 m × 0.25 mm × 0.25 μm, Restek, Bellefonte, Pennsylvania, United States). The temperature program was 60°C (hold 2 min), 30°C/min to 150°C, 10°C/min to 200°C, and 30°C/min to 230°C (hold 5 min). MS conditions were set to 40°C for the manifold, 240°C at the transfer line, and 220°C for the ion source. The scan range was 33–500 m/z for a full scan (scan time 250 ms).

#### 2.4.2 Untargeted metabolomics for nonvolatile metabolites

We analyzed small organic nonvolatile leaf metabolites using untargeted metabolomics following Sedio et al. (2018, 2021). We extracted the samples from ca. 10 mg of homogenized freeze‐dried material using 1.8 mL 90:10 (v/v) methanol/water solvent. We perform the extractions overnight at 4°C and 11 *g*, centrifuged at 24,104 *g* for 30 min, and the supernatant was removed and filtered for analysis using LC–MS. We optimized UHPLC–MS parameters to detect fragment metabolites representing a wide range in polarity and mass (Sedio et al., 2021). We separate the metabolomic extracts using a Thermo Fisher Scientific (Waltham, MA, United States) Vanquish Horizon Duo ultra‐high performance liquid chromatography (UHPLC) system with an Accucore C18 column with 150 mm length, 2.1 mm internal diameter, and 2.6 μm particle size. UHPLC buffer A (0.1% v/v formic acid in water) and buffer B (0.1% v/v formic acid in methanol) were employed in a solvent gradient from 5% to 100% buffer B over 18 min. Metabolites were separated by UHPLC followed by heated electrospray ionization (HESI) in positive mode using full scan MS1 and data‐dependent acquisition of MS2 (dd‐MS2) on a Thermo Fisher Scientific QExactive hybrid quadrupole‐orbitrap mass spectrometer. We analyzed individual willows and quality control (QC) pools, consisting of pooled aliquots for sets of 5 samples. For individuals, we collected an MS1 full scan (115–1725 m/z) at a resolution of 140,000. The MS1 full scan was at 70,000 resolution for pools, followed by dd‐MS2 at 17,500 resolution on the five most abundant precursors found in the MS1 spectrum. Automatic gain control target values were 1e6 for full scan MS1 and 1e5 for dd‐MS2. Maximum ion injection times were 200 ms for full scan MS1, 100 ms for QC MS1, and 50 ms for MS2. For dd‐MS2, we set the isolation window to 1.5 m/z and stepped collision energy at 20, 40, and 60. QC pooled samples were used to account for fluctuations in total ion intensity due to changes in temperature and atmospheric pressure over time.

Raw data from the UHPLC–MS extraction were centroided and processed for peak detection, peak alignment, and peak filtering using Mzmine2 (Pluskal et al., 2010), which groups chromatographic features into putative compounds based on molecular mass and LC retention time. We used the same parameters as Sedio et al. (2021) except for setting the MS1 noise threshold to 15,000 ion count and the MS2 noise threshold to 1500 ion count. We use the MZmine output to calculate metabolite concentrations (peak area per sample dry weight) and putative identities of the metabolites detected. We inferred molecular formulae using Sirius (Dührkop et al., 2019), predicted structures using CSI: finger ID (Dührkop et al., 2015), and classified the metabolites using CANOPUS's ClassyFire compound class predictor (Djoumbou Feunang et al., 2016; Dührkop et al., 2021). We searched the data for salicinoids known to play defensive roles in Salicaceae and additionally included all other metabolites that did not occur in the blanks. All individual metabolite concentrations were standardized as peak area divided by the dry weight of leaf tissue (in mg) and log‐transformed for the analysis.

#### 2.4.3 Targeted proanthocyanidin quantification

We quantified tannins as nonvolatile leaf metabolites with large molecular masses. Proanthocyanidins (condensed tannins) are the major group of tannins in willows (Volf, Hrcek, et al., 2015). We extracted them from ca 20 mg of homogenized material using 1.4 mL of acetone/water (80:20, v/v) solvent as in Malisch et al. (2016). We quantified procyanidin (PC) and prodelphinidin (PD) units found in proanthocyanidins (in mg/g) by UHPLC‐QqQ‐MS/MS following Engström et al. (2014, 2015) as described in, e.g., Malisch et al. (2016). Briefly, we used an Acquity UPLC system (Waters Corp., Milford, MA, USA) coupled with a Xevo TQ triple‐quadrupole mass spectrometer (Waters Corp., Milford, MA, USA). The column used was a 100 mm × 2.1 mm internal diameter, 1.7 μm, Acquity UPLC BEH Phenyl column (Waters Corp., Wexford, Ireland). The flow rate of the eluent was 0.5 mL/min. We use a negative ionization mode for MS analyses. ESI conditions were as follows: capillary voltage, 2.4 kV; desolvation temperature, 650°C; source temperature, 150°C; desolvation and cone gas (N2), 1000 and 100 L/h, respectively; and collision gas, argon. We used purified PC‐rich proanthocyanidin fraction (PC units) and purified PD‐rich proanthocyanidin fraction (PD units) as external standards to quantify these two units of oligomeric and polymeric proanthocyanidins. These results provided the total proanthocyanidin concentration (mg/g dry weight), and mean degree of polymerization (mDP), a critical factor affecting the bioactivity of proanthocyanidins (e.g., Leppä et al., 2020).

### 2.5 Statistical analysis

To test our hypotheses that the specificity in induced plant responses is driven by different herbivore species, order, feeding guild, and level of specialization, we performed multivariate analyses in CANOCO 5 (ter Braak & Smilauer, 2012). We ran separate Redundancy Analyses (RDA) for VOCs and nonvolatile leaf metabolites with plant individuals used as samples and metabolites used as response variables. For the main analysis, we used herbivore order, feeding guild, level of specialization (Table 1), as well as chewing damage (in cm^2^) as explanatory variables. We performed a stepwise forward selection to find the variables that best explained the adjusted variation in VOCs and nonvolatile leaf metabolites. We tested the significance of individual variables and the best overall model with the Monte‐Carlo permutation test with 9999 permutations. Since we did not find any effect from the identity of the two willow individuals from which we obtained the cuttings, we excluded this variable from our analyses.

To further investigate the correlation between insect identity and the observed variation in VOCs and nonvolatile leaf metabolites, we performed a set of individual RDA analyses within Coleoptera and Lepidoptera, the two orders of herbivores with the largest number of species included in our experiment. We used herbivore species as an explanatory variable and performed a stepwise forward selection to explore what species of herbivores would be included in the best model. We performed separate analyses for VOCs and nonvolatile leaf metabolites. We tested the significance of the contribution of the included species towards the explained variation with the Monte‐Carlo permutation test with 9999 permutations. In these analyses, we included herbivore species with both significant and marginally significant effects in the model. Chewing damage was included as a covariable in all analyses. We also performed similar analyses that distinguished between adult leaf beetles and their larvae. As we did not find significant differences between beetle adults and larvae, we merged them in all our analyses.

Additionally, to individually investigate the responses across classes of VOCs and nonvolatile metabolites and tannins among herbivore orders and controls, we perform different sets of variance analysis and post‐hoc tests of multicomparison when the differences observed between treatments were significant. We include supplementary boxplots showing these results for VOCs (Figure S3) and nonvolatile metabolites (Figure S4).

**FIGURE 1:**
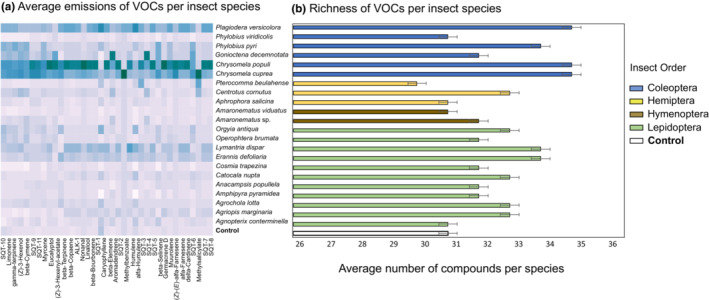
Heatmap plot showing VOC average emissions (area under peaks) (a) and average VOC richness (b) for each insect species and control treatments. The color gradient in panel a shows the emissions of VOCs. Higher emissions are in darker colors, lower emissions are in lighter colors.

**FIGURE 2:**
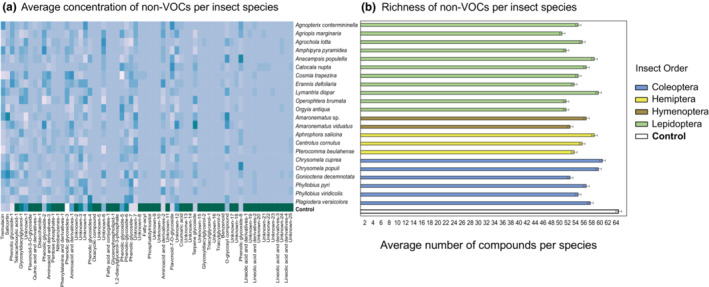
Heatmap plot showing the average concentrations of nonvolatile metabolites (area under peaks) (a) and their average richness (b) for each insect species and control treatments. The color gradient in panel A indicates the concentration of metabolites. Higher concentrations are in darker colors, lower concentrations are in lighter colors.

To further compare the responses in VOCs and nonvolatile leaf metabolites, we calculated the mean concentration of individual compounds in plants exposed to different herbivores and in controls. Using these means, we then calculated a similarity matrix based on the presence and concentration of VOCs or nonvolatile leaf metabolites using the Bray–Curtis similarity index. We tested for the similarity in responses in VOCs and nonvolatile metabolites by correlating the matrices with a complete sample list and each respective value with a Mantel test with 999 permutations in R 4.1.1 (R Core Team, 2021) using the “vegan” package 2.6‐4 (Oksanen et al., 2022). We additionally performed individual Mantel tests—using the same parameters as above—to infer the correlation in responses in VOCs and nonvolatile metabolites using the averaged compound detection per insect order and per insect species. To represent these results, we used heatmaps showing the changes in compound emissions (VOCs, Figure 1a) and concentration (nonvolatile metabolites, Figure 2a) coupled with bar plots presenting the individual changes in compound richness (number of compounds) for VOCs (Figure 1b) and nonvolatile metabolites (Figure 2b) per insect species.

Finally, we fitted linear models to test for the effect of herbivore species, order, feeding guild, and level of specialization on proanthocyanidin concentration and the mean degree of polymerization. We use the herbivore order, feeding guild, level of specialization, and chewing damage as explanatory variables. We also added the effect of plant individuals as a predictor, but since it did not show a significant effect, we excluded it from the models as in the case of multivariate analyses. The effect of individual predictors was tested by analysis of variance, and models were fitted using R 4.1.1.

## 3 RESULTS

### 3.1 Volatile leaf metabolites

**FIGURE 3:**
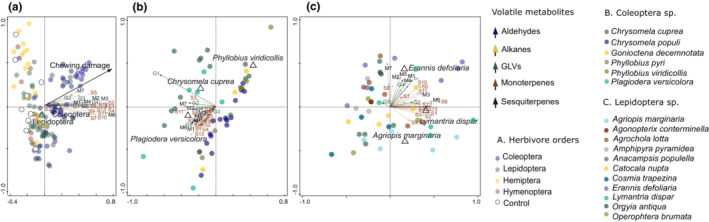
The overall variation in VOC profiles was best explained by chewing damage and insect order (a). Together, chewing damage (pseudo‐*F* = 20.8, *p* = .0001) and the classification of herbivores as Coleoptera (pseudo‐*F* = 57.5, *p* = .0001) or Lepidoptera (pseudo‐*F* = 7.3, *p* = .0027) explained 33.22% of the adjusted variation when analyzed with an RDA with forward selection. The elicited VOC profiles also differed among species within Coleoptera and Lepidoptera. Within Coleoptera, the species that were most correlated with variation in VOC profiles included two specialized leaf beetles *Chrysomela cuprea* (pseudo‐*F* = 3.2, *p* = .0118) and *Plagiodera versicolora* (pseudo‐*F* = 2.6, *p* = .0283), and a generalist weevil *Phyllobius viridicollis* (pseudo‐*F* = 3.3, *p* = .0131). Together, these three species accounted for 12.50% of the adjusted variation in VOC profiles (b). Within Lepidoptera, the species that were most strongly correlated with the variation in VOC profiles included three generalists *Lymantria dispar* (pseudo‐*F* = 3.3, *p* = .0216), *Erannis defoliaria* (pseudo‐*F* = 2.5, *p*‐value = .0506) and *Agriopis marginaria* (pseudo‐*F* = 2.4, *p* = .0563), two of which, however, had only marginally significant effect on the variation in VOCs. The three species jointly explained 11.16% of the adjusted variation in VOCs (c). Individual VOCs are shown as colored arrows, while plant replicates are represented by circles, and color‐coded according to herbivore treatments and controls. The empty triangles represent the centroids (mean point position) of plants damaged by different herbivore species. See Table S1 for full VOC names.

We detected 37 VOCs emitted by the plants upon herbivory. Most of them belonged to sesquiterpenes and monoterpenes, although other classes of VOCs such as green‐leaf‐volatiles (GLVs), alkanes, and aldehydes were also present (Table S1). The overall composition in VOC profiles was best explained by chewing damage and by insect orders (Figure 3a). Together, chewing damage (pseudo‐*F* = 20.8, *p* = .0001) and the classification of herbivores as Coleoptera (pseudo‐*F* = 57.5, *p* = .0001) or Lepidoptera (pseudo‐*F* = 7.3, *p* = .0027) explained 33.22% of the adjusted variation. Meanwhile, the effect of feeding from insects of the orders Hemiptera and Hymenoptera was weaker, and these orders were not selected in the model. Additionally, we recorded no significant effect of herbivore specialization. These results were also reflected by the general trends in the increase of VOCs in plants damaged by different insect orders (Figure S3, Tables S2, S3). Chewing damage by Coleoptera increased the production of several sesquiterpenes as Muurolene, Caryophyllene, and (*Z,E*)‐alfa‐Farnesene and the monoterpene Eucalyptol. The upregulation observed for the other orders was generally weaker, including several compounds that seemingly decreased in response to chewing damage, mostly monoterpenoids and the aldehyde nonanal in plants exposed to Hymenoptera.

The elicited VOCs also differed among species within Coleoptera and Lepidoptera. Within Coleoptera, the species most strongly correlated with variation in VOC profiles included two specialized leaf beetles *Chrysomela cuprea* (pseudo‐*F* = 3.2, *p* = .0118) and *Plagiodera versicolora* (pseudo‐*F* = 2.6, *p* = .0283), as well as a generalist weevil *Phyllobius viridicollis* (pseudo‐*F* = 3.3, *p* = .0131). Together, these three species accounted for 12.50% of the adjusted variation in VOCs among plants exposed to Coleoptera. The VOCs most strongly correlated with feeding by *C. cuprea* included GLVs such as methyl benzoate and methyl salicylate. Feeding by *P. versicolora* was positively correlated with most of the detected sesquiterpenes but more strongly with alfa‐Copaene, Muurolene, beta‐Elemene, Humulene, and delta‐Cadinene, as well as the monoterpene gamma‐Terpinene, the aldehyde nonanal, and an unknown aldehyde. By contrast, most VOCs showed a negative correlation with feeding by *P. viridicollis* (Figure 3b). Within Lepidoptera, the species that best correlated with variation in VOCs included three generalists *Lymantria dispar* (pseudo‐*F* = 3.3, *p* = .0216), *Erannis defoliaria* (pseudo‐*F* = 2.5, *p*‐value = .0506) and *Agriopis marginaria* (pseudo‐*F* = 2.4, *p* = .0563), two of which, however, had only a marginally significant effect on the variation in VOCs. The three species jointly explained 11.16% of the adjusted variation in VOCs among plants exposed to Lepidoptera (Figure 3c). Feeding by *L. dispar* was correlated with the production of sesquiterpenes. Feeding by *E. defoliaria* positively correlated with the monoterpene ϒ‐Terpinene and the GLVs methyl benzoate and methyl salicylate, while feeding by *A. marginaria* generally showed a week or negative correlation to most of the VOCs. We further show the trends observed in VOCs as elicited by different herbivore orders and controls in (Figure S3, Tables S3, S4).

### 3.2 Nonvolatile leaf metabolites

**FIGURE 4:**
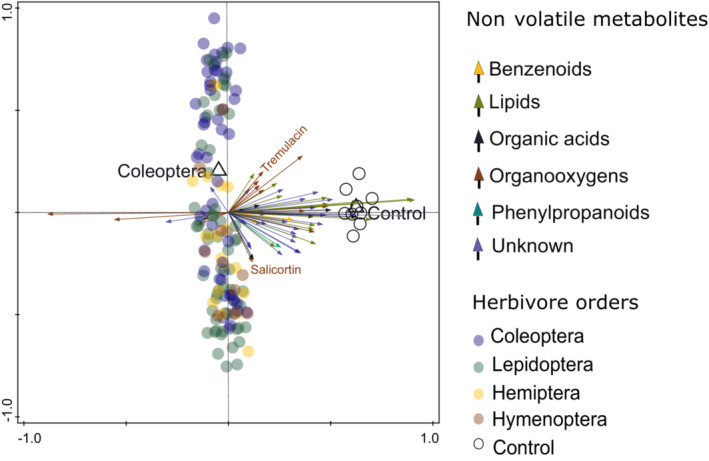
The overall variation in nonvolatile leaf metabolites was best explained by the presence of herbivores when analyzed with an RDA with forward selection. Controls significantly differed from all herbivore treatments (pseudo‐*F* = 18.4, *p*‐value = .0001), explaining 11.49% of the variation. Herbivore identity also played a role to some extent with feeding by Coleoptera explaining 1.5% of the total variation in leaf metabolites (pseudo‐*F* = 2.6, *p* = .0036). Most compounds were downregulated in plants exposed to herbivores. Individual metabolites are shown as colored arrows, while plant replicates are represented by circles, and color‐coded according to herbivore treatments and controls. The empty triangles represent the centroids (mean point position) of plants damaged by different herbivore species.

We detected 64 compounds with untargeted metabolomics when analyzing small nonvolatile leaf metabolites (Table S5). These included both primary and specialized (secondary) metabolites, such as organooxygen compounds, organic acids and derivatives, and lipids and lipid‐like molecules. We also detected benzenoids and phenylpropanoids. Differences in nonvolatile leaf metabolites were best explained by the presence of herbivores, as controls significantly differed from all herbivore treatments (pseudo‐*F* = 17.9, *p*‐value = .0001). The total variation explained was 11.19%, where herbivore identity also played a role to some extent, with feeding by Coleoptera explaining 1.5% of the total variation in leaf metabolites (pseudo‐*F* = 2.6, *p* = .0032; Figure 4). Most compounds were downregulated in the plants exposed to herbivores, including the two detected salicinoids, tremulacin, and salicortin. Only seven organooxygen compounds were upregulated, mostly phenolic glycosides. The overall differences between orders were normally less pronounced than in the case of VOCs, with most nonvolatile metabolites showing lower concentrations in the plants exposed to insects than in the controls (Table S6). The changes in nonvolatiles were not explained by herbivore species when we performed separate analyses in Coleoptera and Lepidoptera. In Lepidoptera, the generalists *E. defoliaria* (pseudo‐*F* = 1.6, *p* = .0552) and *A. marginaria* (pseudo‐*F* = 1.6, *p* = .0708), elicited marginally significant changes that together explained 2.04% of the adjusted variation in nonvolatiles (Figure S2). We further show the trends observed in nonvolatile leaf metabolites as elicited by different herbivore orders and controls in (Table S7).

Finally, the proanthocyanidins and the mean degree of polymerization in the samples did not show any significant response to the treatments (Table S8).

### 3.3 Correlation between volatile and nonvolatile leaf metabolites

There was no correlation between the responses in volatiles and nonvolatile leaf metabolites when we compared the similarity in emissions of metabolites with a Mantel test using every single plant as a separate observation (*R* = 0.044, *p* = .106) or using mean dissimilarities among plants damaged by different insect orders (*R* = 0.030, *p* = .208) and insect species (*R* = 0.123, *p* = .173). We also found no correlation of richness between VOCs and nonvolatile leaf metabolites across herbivore and control treatments (*R* = −0.003, *p* = .485).

## 4 DISCUSSION

Plants employ a great diversity of induced chemical responses to herbivory (Danner et al., 2018; Dicke et al., 2009). Understanding the specificity of plant responses to different herbivore species, levels of specialization, orders, and feeding guilds can help us to reveal some of the factors contributing to the diversity of defensive strategies in plants. Our results suggest that induced plant responses depend on the amount of chewing damage and the identity and order of herbivores. Importantly, the level of specificity differs among VOCs and nonvolatile leaf metabolites that showed largely uncorrelated responses to herbivore identity, providing a further explanation of why there are often different trends in VOCs and nonvolatile defenses among plants (Herrera & Pellmyr, 2002; Zu et al., 2020).

### 4.1 Volatile leaf metabolites

Our results showing that VOC composition was mainly affected by the amount of chewing damage by herbivores (Figure 3a) illustrate that the amount and type of damage are key drivers of the induced response in VOCs (Delphia et al., 2007; Unsicker et al., 2015). Many of the VOCs emitted by plants suffering chewing damage have been shown to have direct and indirect effects on herbivores. For example, emissions of the monoterpene linalool can help reduce subsequent herbivory by reducing herbivore oviposition (De Moraes et al., 2001) or by attracting predators and parasitoids (Kessler & Baldwin, 2001). This is also the case of the GLV (*Z*)‐3‐Hexenyl acetate, which serves as an attractant of natural enemies of herbivores in various Salicaceae (Clavijo McCormick et al., 2014; Peacock et al., 2001). Such a specific induction can theoretically improve the efficacy of similar VOCs against chewing herbivores that induce differential host‐plant defenses and are typically prey to different enemies than sap‐sucking insects (Clavijo McCormick et al., 2012; Mumm & Dicke, 2010).

By contrast, sap‐sucking herbivores tend to induce weaker VOC emissions than chewing herbivores (Danner et al., 2018; Rowen & Kaplan, 2016). Unlike Danner et al. (2018) we did not record any pronounced decrease in VOC emissions in plants damaged by sap‐sucking Hemiptera (Tables S3, S4). These differential trends in VOC profiles as elicited by different insect guilds could be related to triggered metabolic pathways (Rowen & Kaplan, 2016). For instance, sap‐sucking herbivores generally induce the salicylic acid (SA) pathway, whereas leaf‐chewing herbivores tend to elicit the jasmonic acid (JA) pathway (Erb et al., 2012; Walling, 2000). The increase in SA signaling often reduces VOC emission of compounds such as terpenes, which are largely JA‐regulated, especially in response to chewing damage (Schmelzet al., 2003; Wei et al., 2014). We also did not find significant differences among external leaf‐chewing and leaf‐tying chewer herbivores. These two groups can affect the plant differently, for example by changing the photosynthetic capacity of tied leaves, and can have different natural enemies (Nabity et al., 2009; Tvardikova & Novotny, 2012). However, the responses in VOCs in our systems seem to be governed mainly by the type and amount of damage that is largely similar between external leaf‐chewing and leaf‐tying chewer herbivores that both chew on leaves.

In addition to chewing damage, induced responses were also explained by herbivore order (Figure 3a), with Coleoptera (Figure 3b, Figure S3) and Lepidoptera (Figure 3c, Figure S3) inducing different responses in VOCs. Although both orders inflicted chewing damage, lepidopteran larvae chewed on the margins of leaves, while most Coleoptera skeletonized them, which increased the surface of the wound. In combination with the possible specific composition of their oral secretions (Shinya et al., 2016), such differences in feeding mode can contribute to the differential responses this guild elicits. For example, poplars, close relatives of willows, typically show much stronger upregulation of VOCs when damaged by beetles than when damaged by caterpillars (Unsicker et al., 2015). We found a similar pattern here, while we also observed differences among individual species of beetles (Figure 3b, Figure S3). The upregulation of VOCs was particularly strong in the case of the species *Chrysomela cuprea*, which showed strong induction of methyl benzoate, beta‐Terpinene, and Caryophyllene (Figure 3b, Table S2). In comparison, the feeding by *Plagiodera versicolora* was correlated with the induction of methyl salicylate, Linalool, Muurolene, and alfa‐Copaene. Both species are probably well adapted to nonvolatile defenses employed by willows. These specialized beetles can use salicinoids as feeding cues and *C. cuprea* can sequester them (Pasteels et al., 1983; Rank et al., 1998). We did not test the functions of the VOCs elicited by Coleoptera, but several of them, such as (*Z*)‐3‐Hexenyl acetate and methyl salicylate, have been detected in other Salicaceae species (Li et al., 2020; Peacock et al., 2001; Swanson et al., 2021) and can reportedly attract predators in herbaceous plants (Silva et al., 2020). Our results thus suggest that the differential responses in VOCs occur also among plants damaged by different chewing herbivores, which can provide another layer of specificity in the cues to predators and parasitoids feeding nondifferent herbivore orders (Mumm & Dicke, 2010). VOCs could thus constitute an alternative defensive mechanism against the specialized leaf beetles and represent another step in the arms race between willows and their specialized herbivores (Volf, Julkunen‐Tiitto, et al., 2015). Further experiments testing a larger number of replicates and macroevolutionary studies are, however, needed to explore such speculation.

We expected that specialists and generalists would elicit differential responses in VOCs. Differential induction of VOCs among generalist vs. specialist herbivores has been reported, although individual studies provided conflicting results. Sobhy et al. (2017) and Danner et al. (2018) showed higher elicitation of VOCs by generalist herbivores. By contrast, a meta‐analysis by Rowen and Kaplan (2016) found higher VOC induction by specialists. Here we used 10 specialists and 12 species of generalist herbivores. The proportions of generalist and specialist herbivores were not balanced and differed among the studied insect orders, reflecting differences in average specialization among caterpillars, sawflies, beetles, and hemipterans naturally associated with willows (Leong et al., 2022). Our models explaining the variation in VOCs as elicited by Coleoptera (Figure 3b) and Lepidoptera (Figure 3c) included several important generalists and specialist species, such as the specialized leaf beetles or the generalist *Lymantria dispar*. However, the level of specialization as such did not play a significant role in our system. For example, highly specialized Hymenoptera did not elicit different responses than other leaf‐chewing herbivores. Our results thus suggest that although there may be important differences in plant responses induced by species of specialist and generalist herbivores, other traits may be the primary drivers of the response.

### 4.2 Nonvolatile leaf metabolites

There was no correlation between the similarity in responses in VOCs and nonvolatiles. Such differential responses in VOCs and nonvolatile metabolites have been found in earlier studies, and the occurrence of such patterns could be due to the different ecological roles and potential trade‐offs between these two types of defenses (see Gols, 2014). In contrast to VOCs, most of the nonvolatile metabolites detected by untargeted metabolomics showed a largely unspecific response to chewing and sap‐sucking herbivores and were downregulated, with no differentiation between primary and secondary nonvolatile metabolites (Figure S4). This may indicate that the plants redirected the resources from attacked leaves to other organs. Such relocation of metabolites from damaged plant tissues to tissues critical for plant fitness helps plants to protect their resources (Hunziker et al., 2021). Upregulated nonvolatile metabolites included organooxygen compounds, such as phenolic glycosides (Figure 4). Notably, we expected a strong upregulation of salicin and tremulacin, particularly against generalist herbivores. However, the two salicinoids were downregulated in damaged plants. Different salicinoids can show differential responses to herbivory (Fabisch et al., 2019; Fields & Orians, 2006). The strength of the response seems to depend on the leaf age, and it shows less specificity in terms of herbivore identity, or the amount of damage inflicted (Fields & Orians, 2006). Although the changes observed in nonvolatile metabolites differed between control and herbivorized samples, the overall unspecific downregulation of most nonvolatiles suggests that the induced responses we recorded probably do not involve herbivore‐tailored responses. We also did not record any significant response in the degree of polymerization and concentration of proanthocyanidins between herbivore orders (Table S8), possibly because changes in these metabolites with large molecular masses may take longer to occur (Volf et al., 2021). In accordance with other studies, our results thus suggest that induced responses in nonvolatile leaf metabolites may be less specific or occur less rapidly than changes in readily deployable VOCs (Clavijo McCormick et al., 2012; Delphia et al., 2007).

The timing of leaf sampling for nonvolatile quantification may also affect our findings. Plants' volatile and nonvolatile induced responses show pronounced temporal patterns (Mason et al., 2017). For example, some salicinoids can be degraded in damaged leaves over time (Fields & Orians, 2006). It is also important to consider the timing of VOC induction. For instance, the emission of different groups of VOCs showed differential responses to the timing of herbivory in poplars, with GLVs and aldehyde‐derived VOCs emitted shortly after the herbivore attack, while most terpenes were induced as a delayed response to herbivory (Clavijo McCormick et al., 2014). Sampling the VOCs or leaves at different time points would thus likely yield different emissions and concentrations than those recorded here.

## 5 CONCLUSIONS

In conclusion, we found high specificity in VOCs, with much of their composition being explained by the amount of chewing damage, herbivore order, and damage type. Rapidly inducible and specific responses may be particularly relevant in environment‐rich species and for plants that face diverse communities of herbivores and require specific and efficient means of communication (Erb et al., 2012). Here, we provide further evidence of how differential responses to insect herbivores can contribute to the specificity and diversity of plant chemical defensive strategies. Broader studies from multiple plant and herbivore species are required to explore how the specificity in induced responses may contribute to their efficacy and evolutionary trends in plant chemical diversity. For example, there must be some level of conservatism in VOC specificity among co‐occurring or related plants should this specificity constitute reliable cues to predators and parasitoids (Clavijo McCormick et al., 2012; Sobhy et al., 2017). By contrast, high variation in both VOCs and nonvolatile defenses may help co‐occurring‐related plants avoid sharing specialized herbivores (Chen, 2008). Overall, the need to balance these two contrasting pressures may contribute to the variation in defenses between plants and to the differential trends in the evolution of chemical defenses (Zu et al., 2020).

## AUTHOR CONTRIBUTIONS

**Priscila Mezzomo:** Conceptualization (supporting); data curation (lead); formal analysis (lead); methodology (lead); resources (equal); visualization (lead); writing – original draft (lead); writing – review and editing (lead). **Alexander Weinhold:** Data curation (equal); methodology (equal); resources (equal); validation (equal); visualization (equal); writing – review and editing (equal). **Klara Aurova:** Data curation (equal); methodology (equal); writing – review and editing (equal). **Leonardo Re Jorge:** Data curation (equal); formal analysis (equal); investigation (equal); methodology (equal); visualization (equal); writing – review and editing (supporting). **Petr Kozel:** Data curation (equal); investigation (supporting); methodology (equal); writing – review and editing (supporting). **Jan Michalek:** Data curation (equal); investigation (equal); methodology (supporting); writing – review and editing (supporting). **Nela Novakova:** Data curation (equal); methodology (supporting); writing – review and editing (supporting). **Carlo Lutz Seifert:** Conceptualization (equal); data curation (equal); formal analysis (equal); methodology (equal); visualization (equal); writing – review and editing (equal). **Tereza Volfova:** Conceptualization (equal); data curation (equal); investigation (equal); methodology (equal); resources (equal); writing – review and editing (supporting). **Marica Engstrom:** Data curation (equal); methodology (equal); resources (equal); writing – review and editing (supporting). **Juha‐Pekka Salminen:** Data curation (equal); investigation (equal); methodology (equal); resources (equal); writing – review and editing (supporting). **Brian Edward Sedio:** Data curation (equal); formal analysis (equal); investigation (equal); methodology (equal); software (equal); writing – review and editing (equal). **Martin Volf:** Conceptualization (lead); data curation (lead); formal analysis (lead); funding acquisition (lead); investigation (lead); methodology (lead); project administration (lead); resources (lead); supervision (lead); visualization (equal); writing – original draft (lead); writing – review and editing (equal).

## CONFLICT OF INTEREST STATEMENT

There are no conflicts of interest to declare.

## Supporting information


Appendix S1

Appendix S2

Appendix S3

## Data Availability

The authors declare that the data supporting the findings of this study are available within the article and its supplementary information files. The remaining background data are available on Zenodo https://doi.org/10.5281/zenodo.7920258.
