# Supplementary material for: Leaf volatile and nonvolatile metabolites show different levels of specificity in response to herbivory
Source: Ecol Evol. 2023 May 29;13(5):e10123. doi: 10.1002/ece3.10123 (PMC10225982; doi:10.1002/ece3.10123)
Supplement: Supplementary file 1 — Appendix S1 [file ECE3-13-e10123-s002.docx]

**Leaf volatile and non-volatile metabolites show different levels of specificity in response to herbivory**

**Supplementary information – Appendix S1**

Preparation of host plants

After sampling them from the field on February 4^th^, 2020, the cuttings were placed in water for 15 days until they started producing roots. On February 19th, the plants were individually transplanted into 3L plastic pots containing a preparation of soil and sand substrate (4:3). On February 20^th^, we treated the plants with 3% “Prezimující škudci STOP” insecticide (868.5 g/L of *Pongamia pinnata* oil, AGRO, Říkov, CZ) to remove any overwintering herbivores naturally occurring on the cuttings. To remove any remaining insects, we additionally treated the cuttings with 2% Spruzit insecticide (*Pyrethrum roseum* oil, pyrethrins 4.59 g/L, rapeseed oil 825.3 g/L, Neudorf, CZ) on February 25th and March 10th. The plants were grown in the pots for ca 14 weeks before entering the experiment. At first, they were grown in an indoor greenhouse at ca. 20ºC till mid-April. After that, they were moved to another greenhouse where the temperature followed the outdoor conditions. The plants were grown under the natural light regime. They were watered according to necessity.


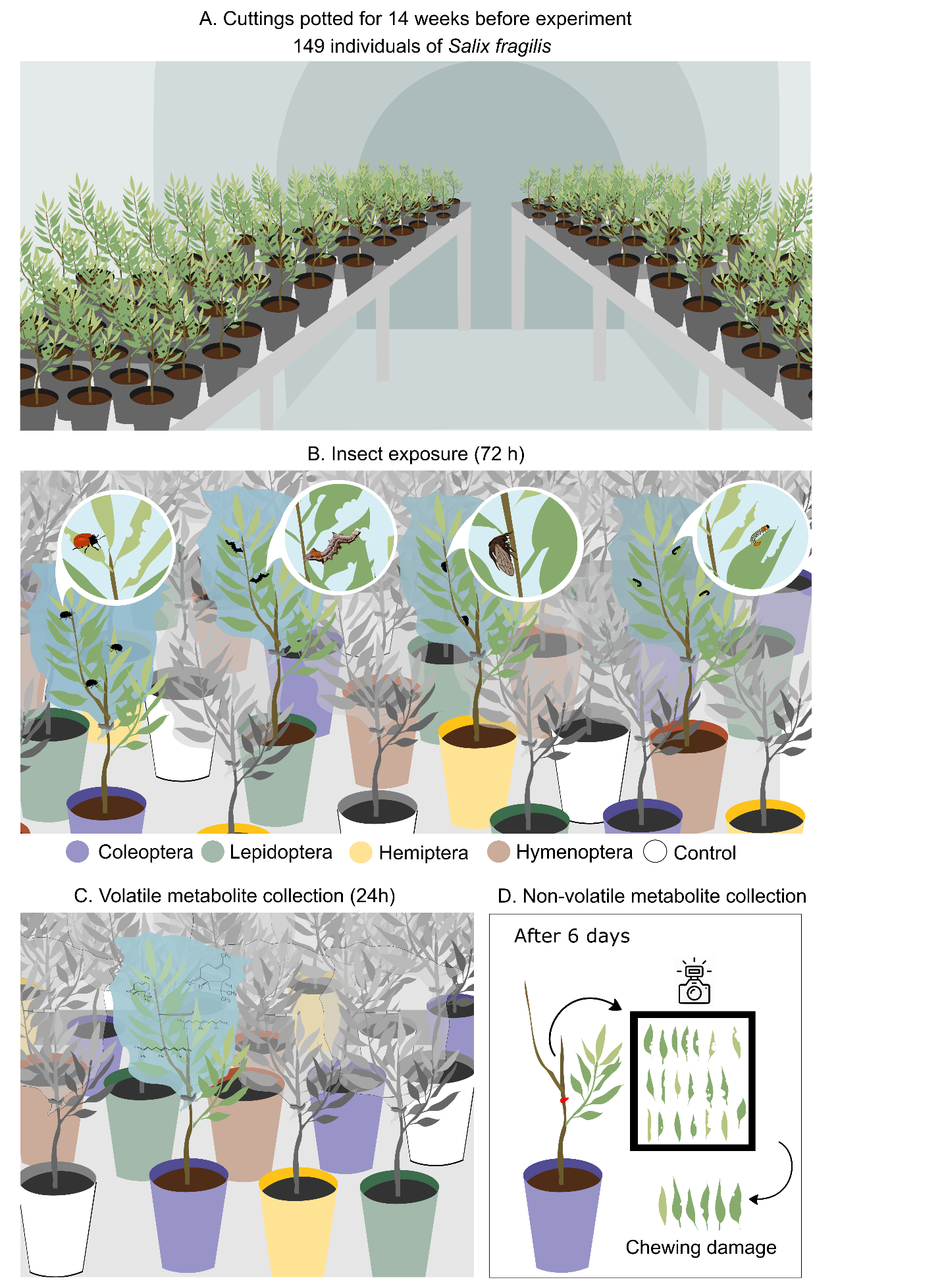


**Figure S1.** We use 149 individuals of *Salix fragilis* in our experiment. The plants were grown in a greenhouse for 14 weeks (A). We introduced the insects to the terminal part of the largest shoot on the plants with ca. 10 leaves and enclosed them in fine mesh transparent tissue bags (B). The insects fed on the plants for 72h and were monitored several times a day. Control plants received bags without herbivores (C). We attached two PDMS (polydimethylsiloxane) tubes to each experimental plant immediately after removing the herbivores and their respective frass and enclosed them in polyamide bags. We passively sample VOCs from headspace for 24 hours. Six days after the VOC collection, we harvested all the leaves that were enclosed in the bags with insects and individually photographed them to measure chewing damage by herbivores. Then, we freeze-dried the first three fully developed upper leaves and homogenized the tissue for further chemical analyses (D).
