# Supplementary material for: Leaf volatile and nonvolatile metabolites show different levels of specificity in response to herbivory
Source: Ecol Evol. 2023 May 29;13(5):e10123. doi: 10.1002/ece3.10123 (PMC10225982; doi:10.1002/ece3.10123)
Supplement: Supplementary file 2 — Appendix S2 [file ECE3-13-e10123-s003.docx]

**Leaf volatile and non-volatile metabolites show different levels of specificity in response to herbivory**

**Supplementary information – Appendix S2**

**Table S1.** VOCs emitted by *Salix fragilis* upon herbivory by 22 species of insect herbivores (Table 1). Compounds are classified according to their class as aldehydes, alkanes, green-leaf-volatiles (GLVs), monoterpenes, and sesquiterpenes. Compound names and legends for identified compounds are presented as shown in Figure 1A-C. Detected retention time (in minutes), measured retention index calculated with a series of n-alkanes (RI ^a^) and retention index based on previous studies using the DB-Wax column according to the NIST library (RI ^b^) are shown in the respective columns.

| Compound class | Compound name | Legend | Retention time (min) | RI ^a^ | RI ^b^ | Ref |
| --- | --- | --- | --- | --- | --- | --- |
| Aldehydes | Nonanal |  | 3.745 | 1408 | 1406 | 1 |
| Alkanes | Alkane 1 |  | 2.103 |  |  |  |
| GLVs | Methyl benzoate | G1 | 5.562 | 1643 | 1641 | 2 |
|  | Methyl salicylate | G2 | 6.572 | 1788 | 1787 | 3 |
|  | (*Z*)-3-Hexenyl acetate | G3 | 3.777 | 1315 | 1315 | 4 |
|  | (*Z*)-3-Hexenol | G4 | 4.078 | 1218 | 1218 | 5 |
| Monoterpenes | ϒ-Terpinene | M1 | 3.433 | 1250 | 1250 | 6 |
|  | β-Cymene | M2 | 3.537 | 1269 | 1267 | 7 |
|  | Linalool | M3 | 4.929 | 1537 | 1534 | 6 |
|  | Limonene | M4 | 2.293 | 1031 | 1031 | 8 |
|  | Myrcene | M5 | 2.979 | 1164 | 1164 | 9 |
|  | Eucalyptol | M6 | 3.204 | 1205 | 1204 | 10 |
|  | β-Terpinene | M7 | 3.266 | 1218 | 1218 | 11 |
| Sesquiterpenes | α-Copaene | S1 | 4.566 | 1470 | 1470 | 12 |
|  | β-Bourbonene | S2 | 4.731 | 1500 | 1500 | 9 |
|  | Caryophyllene | S3 | 5.032 | 1555 | 1554 | 13 |
|  | β-Elemene | S4 | 5.279 | 1598 | 1598 | 14 |
|  | Aromadendrene | S5 | 5.351 | 1610 | 1610 | 15 |
|  | Humulene | S6 | 5.433 | 1623 | 1623 | 16 |
|  | α-Humulene | S7 | 5.516 | 1636 | 1636 | 17 |
|  | β-Selinene | S8 | 5.911 | 1696 | 1695 | 18 |
|  | Germacrene D | S9 | 5.988 | 1707 | 1707 | 19 |
|  | Muurolene | S10 | 6.092 | 1722 | 1722 | 15 |
|  | (*Z, E*)-α-Farnesene | S11 | 6.165 | 1732 | 1732 | 20 |
|  | α-Farnesene | S12 | 6.196 | 1737 | 1737 | 21 |
|  | δ-Cadinene | S13 | 6.294 | 1750 | 1749 | 22 |
|  | Sesquiterpene 1 |  | 5.292 |  |  |  |
|  | Sesquiterpene 2 |  | 5.392 |  |  |  |
|  | Sesquiterpene 3 |  | 5.639 |  |  |  |
|  | Sesquiterpene 4 |  | 5.675 |  |  |  |
|  | Sesquiterpene 5 |  | 5.798 |  |  |  |
|  | Sesquiterpene 6 |  | 6.454 |  |  |  |
|  | Sesquiterpene 7 |  | 6.501 |  |  |  |
|  | Sesquiterpene 8 |  | 6.558 |  |  |  |
|  | Sesquiterpene 9 |  | 6.666 |  |  |  |
|  | Sesquiterpene 10 |  | 6.728 |  |  |  |
|  | Sesquiterpene 11 |  | 6.741 |  |  |  |

*Compounds classified only to the class level are those to which the classification within classes and structure was clear, but the R match was insufficient (<800).

**Table S2.** Emissions (area under the peaks) of individual VOCs by the plants damaged by Coleoptera (COL), Lepidoptera (LEP), Hymenoptera (HYM) or Hemiptera (HEM), and by the controls (CON). Values show the log-transformed average and standard deviation for each compound within the respective treatments.

| Compound class | Compound name | Coleoptera | Lepidoptera | Hymenoptera | Hemiptera | Control |
| --- | --- | --- | --- | --- | --- | --- |
| Aldehydes | Nonanal | 3.29 ± 0.5 | 2.86 ± 0.4 | 2.47 ± 0.2 | 2.58 ± 0.3 | 2.47 ± 0.2 |
| Alkanes | ALK1 | 2.69 ± 0.6 | 2.29 ± 0.5 | 1.92 ± 0.2 | 1.62 ± 0.9 | 1.49 ± 0.8 |
| GLVs | (*Z*)-3-Hexenol | 3.03 ± 0.4 | 2.72 ± 0.2 | 2.61 ± 0.2 | 2.69 ± 0.3 | 2.61 ± 0.2 |
|  | (*Z*)-3-Hexenyl-acetate | 2.99 ± 0.6 | 2.57 ± 0.4 | 2.56 ± 0.4 | 2.54 ± 0.3 | 2.33 ± 0.4 |
|  | Methylbenzoate | 0.82 ± 1.4 | 0.00 ± 0.0 | 0.29 ± 0.7 | 0.00 ± 0.0 | 0.00 ± 0.0 |
|  | Methylsalicylate | 2.89 ± 0.5 | 2.58 ± 0.3 | 2.42 ± 0.3 | 2.80 ± 0.6 | 2.36 ± 0.2 |
| Monoterpenes | Eucalyptol | 4.80 ± 0.5 | 4.21 ± 0.5 | 3.80 ± 0.3 | 3.70 ± 0.4 | 3.54 ± 0.3 |
|  | ϒ-Terpinene | 2.87 ± 0.3 | 2.62 ± 0.2 | 2.49 ± 0.2 | 2.59 ± 0.2 | 2.55 ± 0.2 |
|  | Limonene | 3.77 ± 0.6 | 3.51 ± 0.4 | 3.40 ± 0.2 | 3.51 ± 0.8 | 3.46 ± 0.4 |
|  | Linalool | 3.79 ± 0.4 | 3.30 ± 0.3 | 3.07 ± 0.6 | 2.87 ± 0.8 | 2.88 ± 0.6 |
|  | Muurolene | 3.65 ± 0.3 | 3.25 ± 0.2 | 2.71 ± 0.2 | 2.82 ± 0.2 | 2.84 ± 0.2 |
|  | Myrcene | 2.62 ± 0.3 | 2.36 ± 0.3 | 2.27 ± 0.2 | 2.36 ± 0.2 | 2.26 ± 0.3 |
| Sesquiterpenes | (*Z,E*)-α-Farnesene | 4.77 ± 0.7 | 3.91 ± 0.6 | 3.52 ± 0.5 | 3.52 ± 0.5 | 2.83 ± 0.4 |
|  | α-Copaene | 3.51 ± 0.5 | 3.03 ± 0.4 | 2.58 ± 0.3 | 2.62 ± 0.3 | 2.52 ± 0.2 |
|  | α-Farnesene | 3.59 ± 0.5 | 3.11 ± 0.4 | 2.69 ± 0.2 | 2.74 ± 0.3 | 2.61 ± 0.2 |
|  | α-Humulene | 3.73 ± 0.4 | 3.56 ± 0.6 | 3.49 ± 0.2 | 3.61 ± 0.3 | 3.55 ± 0.4 |
|  | Aromadendrene | 4.70 ± 0.4 | 4.47 ± 0.3 | 4.37 ± 0.3 | 4.54 ± 0.3 | 4.29 ± 0.2 |
|  | β-Bourbonene | 3.30 ± 0.5 | 2.81 ± 0.4 | 2.42 ± 0.2 | 2.48 ± 0.3 | 2.33 ± 0.2 |
|  | β-Cymene | 2.79 ± 0.3 | 2.52 ± 0.3 | 2.44 ± 0.3 | 2.48 ± 0.2 | 2.44 ± 0.2 |
|  | β-Elemene | 3.51 ± 0.5 | 3.01 ± 0.5 | 2.55 ± 0.2 | 2.60 ± 0.3 | 2.49 ± 0.3 |
|  | β-Selinene | 3.69 ± 0.4 | 3.39 ± 0.3 | 3.33 ± 0.2 | 3.39 ± 0.3 | 3.29 ± 0.4 |
|  | β-Terpinene | 1.34 ± 1.2 | 0.81 ± 1.0 | 0.52 ± 0.8 | 0.93 ± 0.9 | 0.36 ± 0.7 |
|  | Caryophyllene | 2.76 ± 0.3 | 2.67 ± 0.4 | 2.55 ± 0.2 | 2.69 ± 0.3 | 2.66 ± 0.5 |
|  | δ-Cadinene | 3.46 ± 0.5 | 2.99 ± 0.4 | 2.53 ± 0.2 | 2.56 ± 0.3 | 2.46 ± 0.2 |
|  | Germacrene-D | 3.49 ± 1.0 | 2.46 ± 1.3 | 1.50 ± 1.2 | 1.29 ± 1.5 | 1.25 ± 1.3 |
|  | Humulene | 1.60 ± 1.4 | 0.96 ± 1.3 | 0.00 ± 0.0 | 0.14 ± 0.6 | 0.19 ± 0.5 |
|  | SQT-1 | 2.95 ± 0.5 | 2.48 ± 0.4 | 2.07 ± 0.2 | 2.18 ± 0.3 | 2.04 ± 0.2 |
|  | SQT-2 | 1.60 ± 1.4 | 0.59 ± 1.1 | 0.00 ± 0.0 | 0.14 ± 0.6 | 0.20 ± 0.6 |
|  | SQT-3 | 1.65 ± 1.4 | 0.82 ± 1.1 | 0.00 ± 0.0 | 0.62 ± 1.2 | 0.19 ± 0.5 |
|  | SQT-4 | 4.33 ± 0.4 | 4.08 ± 0.3 | 3.97 ± 0.3 | 4.15 ± 0.3 | 3.89 ± 0.2 |
|  | SQT-5 | 3.51 ± 0.5 | 3.05 ± 0.4 | 2.57 ± 0.1 | 2.64 ± 0.3 | 2.53 ± 0.2 |
|  | SQT-6 | 3.05 ± 0.4 | 2.74 ± 0.3 | 2.66 ± 0.3 | 2.72 ± 0.2 | 2.64 ± 0.3 |
|  | SQT-7 | 2.87 ± 0.8 | 2.27 ± 0.8 | 1.19 ± 1.0 | 1.33 ± 1.1 | 1.34 ± 1.0 |
|  | SQT-8 | 2.72 ± 0.9 | 1.99 ± 1.0 | 1.22 ± 1.1 | 1.01 ± 1.1 | 1.35 ± 1.0 |
|  | SQT-9 | 3.91 ± 0.5 | 3.46 ± 0.3 | 3.26 ± 0.3 | 3.41 ± 0.4 | 3.15 ± 0.3 |
|  | SQT-10 | 3.57 ± 0.3 | 3.31 ± 0.3 | 3.20 ± 0.2 | 3.32 ± 0.2 | 3.34 ± 0.3 |
|  | SQT-11 | 5.17 ± 0.4 | 4.75 ± 0.4 | 4.55 ± 0.4 | 4.66 ± 0.4 | 4.36 ± 0.4 |

**Table S3.** Emissions (area under the peaks) of monoterpenes, sesquiterpenes, green-leaf-volatiles (GLVs), aldehydes, and alkanes by the plants damaged by Coleoptera (COL), Lepidoptera (LEP), Hymenoptera (HYM) and Hemiptera (HEM), and by the controls (CON). Values show the log-transformed average, standard deviation, and significance values from the variance analysis for each compound class within the insect orders and control treatments. Asterisks show significance.

|  | Monoterpenes | | Sesquiterpenes | | GLVs | | |
| --- | --- | --- | --- | --- | --- | --- | --- |
| Groups | Mean ± std | p | Mean ± std | p | Mean ± std | | p |
| COL | 3.16 ± 1.18 | 0.000*** | 3.28 ± 1.09 | 0.000*** | 2.97 ± 1.74 | | 0.000*** |
| LEP | 2.78 ± 1.11 | 0.000*** | 2.79 ± 1.15 | 0.000*** | 2.47 ± 1.71 | | 0.000*** |
| HYM | 2.59 ± 1.05 | 0.000*** | 2.37 ± 1.25 | 0.000*** | 2.46 ± 1.58 | | 0.002** |
| HEM | 2.46 ± 1.31 | 0.000*** | 2.65 ± 0.99 | 0.000*** | 2.50 ± 1.70 | | 0.001*** |
| CON | 2.51 ± 1.05 | 0.000*** | 2.35 ± 1.20 | 0.000*** | 2.26 ± 1.57 | | 0.000*** |
| Aldehydes | | | | Alkanes | | | |
| Groups | Mean ± std | p | | Mean ± std | | P | |
| COL | 3.29 ± 0.46 | 0.000*** | | 2.69 ± 0.60 | | 0.000*** | |
| LEP | 2.86 ± 0.35 | 0.000*** | | 2.29 ± 0.48 | | 0.000*** | |
| HYM | 2.47 ± 0.19 | 0.000*** | | 1.92 ± 0.18 | | 0.000*** | |
| HEM | 2.58 ± 0.26 | 0.000*** | | 1.62 ± 0.89 | | 0.003** | |
| CON | 2.47 ± 0.20 | 0.000*** | | 1.49 ± 0.01 | | 0.003** | |

Significance codes: 0 ‘***’ 0.001 ‘**’ 0.01 ‘*’ 0.05 ‘.’ 0.1 ‘ ’ 1

**Table S4.** Post-hoc test results for emissions (area under the peaks) of monoterpenes, sesquiterpenes, green-leaf-volatiles (GLVs), aldehydes, and alkanes by the plants damaged by Coleoptera (COL), Lepidoptera (LEP), Hymenoptera (HYM) and Hemiptera (HEM), and by the controls (CON). Values show the estimate, standard error, t-value and significance values for each compound class within the insect orders and control treatments. Asterisks show significance.

|  | Monoterpenes | | | | Sesquiterpenes | | | |
| --- | --- | --- | --- | --- | --- | --- | --- | --- |
| Groups | Estimate | Std | t-value | p | Estimate | Std | t-value | p  Estimate |
| COL-CON | -0.63 | 0.11 | -5.77 | <0.001*** | -0.65 | 0.11 | -6.14 | <0.001*** |
| COL-HEM | -0.54 | 0.08 | -6.45 | <0.001*** | -0.49 | 0.08 | -6.04 | <0.001*** |
| COL-HYM | -0.56 | 0.12 | -4.62 | <0.001*** | -0.58 | 0.12 | -4.90 | <0.001*** |
| COL-LEP | -0.37 | 0.06 | -5.98 | <0.001*** | -0.35 | 0.06 | -5.80 | <0.001*** |
| CON-HEM | 0.09 | 0.12 | 0.77 | 0.935 | 0.16 | 0.12 | 1.38 | 0.6232 |
| CON-HYM | 0.07 | 0.15 | 0.45 | 0.991 | 0.07 | 0.15 | 0.49 | 0.9868 |
| CON-LEP | 0.26 | 0.11 | 2.36 | 0.123 | 0.30 | 0.11 | 2.84 | 0.0377* |
| HEM-HYM | -0.03 | 0.13 | -0.20 | 1.00 | -0.09 | 0.13 | -0.71 | 0.9507 |
| HEM-LEP | 0.17 | 0.08 | 1.97 | 0.269 | 0.14 | 0.08 | 1.70 | 0.420 |
| HYM-LEP | 0.19 | 0.12 | 1.57 | 0.502 | 0.23 | 0.12 | 1.94 | 0.2849 |
|  | GLVs | | | | Aldehydes | | | |
| Groups | Estimate | Std | t-value | p | Estimate | Std | t-value | p  Estimate |
| COL-CON | -0.53 | 0.12 | -4.47 | < 0.001*** | -0.81 | 0.14 | -5.91 | <0.001*** |
| COL-HEM | -0.28 | 0.09 | -3.11 | 0.017* | -0.71 | 0.10 | -6.75 | <0.001*** |
| COL-HYM | -0.45 | 0.13 | -3.40 | 0.007** | -0.82 | 0.15 | -5.35 | <0.001*** |
| COL-LEP | -0.33 | 0.07 | -5.00 | < 0.001*** | -0.42 | 0.08 | -5.41 | <0.001*** |
| CON-HEM | 0.25 | 0.13 | 1.87 | 0.320 | 0.11 | 0.15 | 0.69 | 0.956 |
| CON-HYM | 0.08 | 0.16 | 0.49 | 0.987 | -0.01 | 0.19 | -0.04 | 1.000 |
| CON-LEP | 0.19 | 0.12 | 1.63 | 0.463 | 0.39 | 0.14 | 2.82 | 0.040* |
| HEM-HYM | -0.17 | 0.14 | -1.16 | 0.760 | -0.11 | 0.17 | -0.67 | 0.959 |
| HEM-LEP | -0.06 | 0.09 | -0.62 | 0.970 | 0.28 | 0.11 | 2.69 | 0.056. |
| HYM-LEP | 0.11 | 0.13 | 0.85 | 0.907 | 0.40 | 0.15 | 2.58 | 0.074. |
|  | Alkanes | | | |  | | | |
| Groups | Estimate | Std | t-value | p |  |  |  |  |
| COL-CON | -1.20 | 0.23 | -5.25 | < 0.001*** |  |  |  |  |
| COL-HEM | -1.07 | 0.17 | -6.18 | < 0.001*** |  |  |  |  |
| COL-HYM | -0.77 | 0.25 | -3.02 | 0.023* |  |  |  |  |
| COL-LEP | -0.40 | 0.13 | -3.06 | 0.021* |  |  |  |  |
| CON-HEM | 0.13 | 0.26 | 0.49 | 0.987 |  |  |  |  |
| CON-HYM | 0.43 | 0.32 | 1.36 | 0.637 |  |  |  |  |
| CON-LEP | 0.80 | 0.23 | 3.50 | 0.005** |  |  |  |  |
| HEM-HYM | 0.30 | 0.28 | 1.09 | 0.800 |  |  |  |  |
| HEM-LEP | 0.68 | 0.17 | 3.88 | 0.001** |  |  |  |  |
| HYM-LEP | 0.37 | 0.25 | 1.46 | 0.571 |  |  |  |  |

Significance codes: 0 ‘***’ 0.001 ‘**’ 0.01 ‘*’ 0.05 ‘.’ 0.1 ‘ ’ 1

**Table S5.** Non-volatile metabolites detected in *Salix fragilis* after herbivory by 22 species of insect herbivores (Table 1). Compounds were analyzed by UHPLC-MS and untargeted metabolomics. They are divided according to their superclass into benzenoids, lipids and lipid-like molecules, organic acids, and derivatives, organooxygen compounds, phenylpropanoids and polyketides, and unknown compounds as the ones to which there were no library matches. Compound putative names are described according to the most specific class from processing output, ion mass (mass to charge ratio), and retention times in seconds.

| Compound superclass | Compound putative name | Ion mass | Retention time (sec) |
| --- | --- | --- | --- |
| Benzenoids | Benzenoid 1 | 741.533 | 1032.510 |
|  | Nitrobenzenes 1 | 301.065 | 548.022 |
| Lipids and lipid-like molecules | 1,2-diacylglycerol-3-phosphates | 717.446 | 1108.640 |
|  | Fatty acid and conjugates 1 | 371.163 | 1171.245 |
|  | Fatty acyl 1 | 223.130 | 518.426 |
|  | Glycerophospholipid 1 | 717.447 | 1142.210 |
|  | Glycosyldiacylglycerol 1 | 839.497 | 1007.970 |
|  | Glycosyldiacylglycerol 2 | 839.497 | 1007.230 |
|  | Lineolic acid and derivatives 1 | 595.471 | 999.031 |
|  | Lineolic acid and derivatives 2 | 335.220 | 759.454 |
|  | Lineolic acid and derivatives 3 | 595.471 | 991.232 |
|  | Lineolic acid and derivatives 4 | 595.472 | 970.355 |
|  | Phosphatidylinositol 1 | 855.499 | 1089.270 |
|  | Terpene glycoside 1 | 447.209 | 965.390 |
|  | Triacylglycerol 1 | 875.709 | 1261.230 |
|  | Triacylglycerol 2 | 899.709 | 1074.680 |
| Organic acids and derivatives | Amino acid and derivatives 1 | 569.162 | 628.249 |
|  | Amino acid and derivatives 2 | 465.136 | 450.208 |
|  | Amino acid and derivatives 3 | 419.177 | 952.814 |
|  | Phenylalanine and derivatives 1 | 465.136 | 457.627 |
|  | Tetracarboxylic acid 1 | 689.183 | 674.677 |
| Organooxygen compounds | Disaccharide 1 | 617.183 | 552.511 |
|  | Tremulacin | 551.152 | 643.160 |
|  | O-glycosyl compound 1 | 861.477 | 1091.270 |
|  | Oxacyclic compound 1 | 200.044 | 36.727 |
|  | Pentose phosphate 1 | 567.125 | 636.437 |
|  | Salicortin | 447.126 | 456.078 |
|  | Phenolic glycoside 1 | 585.157 | 538.816 |
|  | Phenolic glycoside 2 | 617.183 | 564.009 |
|  | Phenolic glycoside 3 | 585.158 | 528.521 |
|  | Phenolic glycoside 4 | 389.123 | 547.622 |
|  | Phenolic glycoside 5 | 617.183 | 558.340 |
|  | Phenolic glycoside 6 | 617.183 | 567.475 |
|  | Phenolic glycoside 7 | 349.089 | 343.645 |
|  | Phenolic glycoside 8 | 585.157 | 547.760 |
|  | Quinic acid and derivatives 1 | 377.084 | 213.588 |
| Phenylpropanoids and polyketides | Coumaric acid 1 | 455.027 | 317.105 |
|  | Flavonoid-3-O-glycoside 1 | 595.165 | 491.623 |
|  | Flavonoid-7-O-glycoside 1 | 617.147 | 491.884 |
| Unknown | Unknown 1-25 |  |  |

**Table S6.** Concentration (area under the peaks/mg) of benzenoids, lipids and lipid-like molecules, organic acids, and derivatives, organooxygen compounds, phenylpropanoids and polyketides, and unknown compounds in the plants damaged by Coleoptera (COL), Lepidoptera (LEP), Hymenoptera (HYM) or Hemiptera (HEM), and in the controls (CON). Values show the log-transformed average and standard deviation for each compound class within the insect orders and control treatments. Asterisks show significance.

|  | Benzenoids | | Lipids | | Organic acids | |
| --- | --- | --- | --- | --- | --- | --- |
| Groups | Mean ± std | p | Mean ± std | p | Mean ± std | p |
| COL | 2.18 ± 3.27 | <0.001*** | 1.52 ± 2.99 | <0.001*** | 6.96 ± 3.39 | <0.001*** |
| CON | 2.69 ± 3.37 | 0.001** | 1.25 ± 2.78 | <0.001*** | 6.88 ± 3.39 | 0.000*** |
| HEM | 2.85 ± 3.17 | 0.564 | 1.06 ± 2.70 | 0.436 | 7.13 ± 3.08 | 0.450 |
| HYM | 2.88 ± 3.34 | 0.120 | 1.21 ± 2.78 | 0.484 | 6.74 ± 3.48 | 0.279 |
| LEP | 6.59 ± 2.81 | 0.824 | 6.50 ± 3.09 | 0.984 | 8.75 ± 0.90 | 0.071. |
|  | Organooxygen**s** | | Phenylpropanoids | | Unknown | |
| Groups | Mean ± std | p | Mean ± std | p | Mean ± std | p |
| COL | 5.69 ± 4.15 | < 0.001*** | 5.24 ± 3.73 | <0.001*** | 3.10 ± 3.62 | <0.001*** |
| CON | 5.29 ± 4.29 | 0.000 *** | 6.34 ± 3.31 | 0.000*** | 3.43 ± 3.62 | <0.001*** |
| HEM | 5.56 ± 4.21 | 0.065. | 5.80 ± 3.51 | 0.285 | 3.71 ± 3.57 | 0.351 |
| HYM | 5.34 ± 4.22 | 0.938 | 6.13 ± 3.38 | 0.786 | 3.30 ± 3.60 | 0.031* |
| LEP | 6.96 ± 3.52 | 0.010* | 8.42 ± 1.03 | 0.029* | 6.25 ± 3.22 | 0.026* |

Significance codes: 0 ‘***’ 0.001 ‘**’ 0.01 ‘*’ 0.05 ‘.’ 0.1 ‘ ’ 1

**Table S7**. Post-hoc test for concentration (area under the peaks/mg) of benzenoids, lipids and lipid-like molecules, organic acids and derivatives, organooxygen compounds, phenylpropanoids and polyketides, and unknown compounds in the plants damaged by Coleoptera (COL), Lepidoptera (LEP), Hymenoptera (HYM) or Hemiptera (HEM), and in the controls (CON). Values show the estimate, standard error, t-value and significance values for each compound class within the insect orders and control treatments. Asterisks show significance.

|  | Benzenoids | | | | Lipids and lipid-like molecules | | | |
| --- | --- | --- | --- | --- | --- | --- | --- | --- |
| Groups | Estimate | Std | t-value | p | Estimate | Std | t-value | p  Estimate |
| COL-CON | 2.41 | 0.73 | 3.27 | 0.010* | 4.91 | 0.26 | 18.74 | <0.001*** |
|  | Organic acids and derivatives | | | | Organooxygen compounds | | | |
| Groups | Estimate | Std | t-value | p | Estimate | Std | t-value | p  Estimate |
| COL-CON | 1.19 | 0.33 | 3.58 | 0.004** | 1.31 | 0.27 | 4.82 | < 0.001*** |
| COL-HEM | - | - | - | - | -0.38 | 0.21 | -1.86 | 0.325 |
| COL-LEP | 0.31 | 0.17 | 1.82 | 0.348 | -0.37 | 0.14 | -2.60 | 0.070. |
| CON-HEM | - | - | - | - | -1.70 | 0.31 | -5.49 | < 0.001*** |
| CON-LEP | -0.87 | 0.33 | -2.65 | 0.061. | -1.68 | 0.27 | -6.21 | < 0.001*** |
| HEM-LEP | - | - | - | - | 0.01 | 0.20 | 0.07 | 1.000 |
|  | Phenylpropanoids and polyketides | | | | Unknown compounds | | | |
| Groups | Estimate | Std | t-value | p | Estimate | Std | t-value | p |
| COL-CON | 2.79 | 0.61 | 4.60 | < 0.001*** | 3.11 | 0.28 | 11.27 | <0.001*** |
| COL-HYM | - | - | - | - | 0.60 | 0.28 | 2.18 | 0.181 |
| COL-LEP | 0.70 | 0.32 | 2.21 | 0.168 | 0.32 | 0.14 | 2.25 | 0.156 |
| CON-HYM | - | - | - | - | -2.51 | 0.36 | -6.95 | <0.001*** |
| CON-LEP | -2.09 | 0.60 | -3.46 | 0.006** | -2.79 | 0.27 | -10.16 | <0.001*** |
| HYM-LEP | - | - | - | - | -0.28 | 0.27 | -1.01 | 0.841 |

Significance codes: 0 ‘***’ 0.001 ‘**’ 0.01 ‘*’ 0.05 ‘.’ 0.1 ‘ ’ 1

**Table S8**. Concentration of proanthocyanidins (mg/g) in the plants herbivorized by Coleoptera (COL), Lepidoptera (LEP), and Hymenoptera (HYM) and Hemiptera (HEM), and in the controls (CON). Values show the treatments' averages, standard deviations, and significance values for each compound class within the insect orders and control treatments.

| Proanthocyanidin (mg/g) | | | Mean degree of polymerization (mDP) | |
| --- | --- | --- | --- | --- |
| Groups | Mean ± Std | p | Mean ± std | p |
| COL | 2.33 ± 1.63 | 0.555 | 3.22 ± 0.93 | 0.3617 |
| LEP | 2.44 ± 1.50 | 0.983 | 3.16 ± 0.92 | 0.101 |
| HYM | 2.13 ± 1.92 | 0.690 | 3.03 ± 0.86 | 0.300 |
| HEM | 2.67 ± 2.41 | 0.109 | 3.01 ± 1.02 | 0.179 |
| CON | 2.09 ± 1.44 | 0.903 | 2.56 ± 0.86 | 0.555 |

**References – Table S1**

1. Mahajan, S.S., Goddik, L., Qian, M.C. (2004) Aroma Compounds in Sweet Whey Powder. *Journal of Dairy Science, 87(12)*, 4057-4063, https://doi.org/10.3168/jds.S0022-0302(04)73547-X

2. Wei A., Shibamoto T. (2007) Antioxidant activities and volatile constituents of various essential oils. *Journal of Agricultural and Food Chemistry, 55(5)*, 1737-1742, https://doi.org/10.1021/jf062959x

3. Coen, M., Engel, R., Nahrstedt, A. (1995) Chavicol β-D-glucoside, a phenylpropanoid heteroside, benzyl-β-D-glucoside and glycosidically bound volatiles from subspecies of *Cedronella canariensis*. *Phytochemistry, 40(1),* 149-155. https://doi.org/10.1016/0031-9422(95)00241-X

4. Engel, K.-H., Flath, R.A.; Buttery, R.G., Mon, T.R., Ramming, D.W., Teranishi, R. (1988). Investigation of volatile constituents in nectarines: Analytical and sensory characterization of aroma components in some nectarine cultivars. *Journal of Agricultural and Food Chemistry, 36(3),* 549-553, https://doi.org/10.1021/jf00081a036

5. Zheng, C.H., Kim, K.H., Kim, T.H., Lee, H.J. (2005) Analysis and characterization of aroma-active compounds of *Schizandra chinensis* (omija) leaves. *Journal of Agricultural and Food Chemistry, 85(1),* 161-166, https://doi.org/10.1002/jsfa.1975

6. Osorio, C., Alarcon, M., Moreno, C., Bonilla, A., Barrios, J., Garzon, C., Duque, C. (2006) Characterization of Odor-Active Volatiles in Champa (*Campomanesia lineatifolia* R.P.). *Journal of Agricultural and Food Chemistry, 54(2)*, 509-516, https://doi.org/10.1021/jf052098c

7. Le Quere JL, Latrasse A. (1990). Composition of the Essential Oils of Blackcurrant Buds (*Ribes nigrum* L.). *Journal of Agricultural and Food Chemistry, 38(1)*, 3-10. https://doi.org/10.1021/jf00091a001

8. Nogueira, P.C.L.; Bittrich, V.; Shepherd, G.J.; Lopes, A.V.; Marsaioli, A.J. (2001) The ecological and taxonomic importance of flower volatiles of *Clusia* species (Guttiferae). *Phytochemistry, 56(5),* 443-452, https://doi.org/10.1016/S0031-9422(00)00213-2

9. Lopes, D., Strobl, H., Kolodziejczyk, P. (2004) 14-Methylpentadecano-15-lactone (Muscolide): a new macrocyclic lactone from the oil of *Angelica archangelica* L., *Chemistry and Biodiversity, 1(12)*, 1880-1887, https://doi.org/10.1002/cbdv.200490144

10. Ruiz Perez-Cacho, P., Mahattanatawee, K., Smoot, J.M., Rouseff, R. (2007) Identification of Sulfur Volatiles in Canned Orange Juices Lacking Orange Flavor. *Journal of Agricultural and Food Chemistry, 55(14)*, 5761-5767, https://doi.org/10.1021/jf0703856

11. Zeng, Z., Xie, R., Zhang, T., Zhang, H., Chen, J.Y. (2011) Analysis of volatile compositions of *Magnolia biondii* Pamp by steam distillation and Headspace solid phase micro-extraction. *Journal of Oleo Science, 60(12),* 591-596, https://doi.org/10.5650/jos.60.591

12. Fakhari A.R., Sonboli A., Heydari R. (2005) Composition of the essential oil of *Rhabdosciadium strausii* from Iran. *Chemistry of Natural Compounds, 41(4)*, 413-414, https://doi.org/10.1007/s10600-005-0164-1

13. Franco, M.R.B., Shibamoto, T. (2000). Volatile composition of some Brazilian fruits: umbu-caja (*Spondias citherea*), camu-camu (*Myrciaria dubia*), araca-boi (*Eugenia stipitata*), and cupuacu (*Theobroma grandiflorum)*. *Journal of Agricultural and Food Chemistry, 48(4)*, 1263-1265, https://doi.org/10.1021/jf9900074

14. Ngassoum, M.B., Yonkeu, S., Jirovetz, L., Buchbauer, G., Schmaus, G., Hammerschmidt, F.-J.H. (1999) Chemical composition of essential oils of *Lantana camara* leaves and flowers from Cameroon and Madagascar. *Flavour and Fragrance Journal, 14(4)*, 245-250, https://doi.org/10.1002/(SICI)1099-1026(199907/08)14:4<245::AID-FFJ819>3.0.CO;2-X

15. Umano, K., Hagi, Y., Nakahara, K., Shoji, A., Shibamoto, T. (2000) Volatile chemicals identified in extracts from leaves of Japanese mugwort (*Artemisia princeps* Pamp.). *Journal of Agricultural and Food Chemistry, 48(8)*, 3463-3469, https://doi.org/10.1021/jf0001738

16. Giuseppe, Z., Manuela, G., Marta, B., Vincenzo, G. (2005) Application of artificial neural network on mono- and sesquiterpenes compounds determined by headspace solid-phase microextraction-gas chromatography-mass spectrometry for the Piedmont ricotta cheese traceability. *Journal of Chromatography A1071(1-2)*, 247-253, https://doi.org/10.1016/j.chroma.2004.11.083

17. Nébié, R.H.C., Yaméogo, R.T., Bélanger, A., Sib, F.S. (2004) Composition chimique des huiles essentielles d'Ageratum conyzoïdes du Burkina Faso, *Comptes Rendus Chimie, 7,* 10-11, 1019-1022, https://doi.org/10.1016/j.crci.2003.12.027

18. Choi, H.-S., Kim. M.-S.L., Sawamura, M. (2002) Constituents of the essential oil of *Cnidium officinale* Makino, a Korean medicinal plant. *Flavour and Fragrance Journal, 17(1)*, 49-53, https://doi.org/10.1002/ffj.1038

19. Kim, T.H., Thuy, N.T., Shin, J.H., Baek, H.H., Lee, H.J. (2000) Aroma-active compounds of miniature beefsteak plant (*Mosla dianthera* Maxim.). *Journal of Agricultural and Food Chemistry, 48(7)*, 2877-2881, https://doi.org/10.1021/jf000219x

20. Seo, W.H., Baek, H.H. (2005) Identification of characteristic aroma-active compounds from water dropword (*Oenanthe javanica* DC.). *Journal of Agricultural and Food Chemistry, 53(17)*, 6766-6770, https://doi.org/10.1021/jf050150z

21. Ferrari, G., Lablanquie, O., Cantagrel, R., Ledauphin, J., Payot, T., Fournier, N., Guichard, E. (2004) Determination of key odorant compounds in freshly distilled cognac using GC-O, GC-MS, and sensory evaluation. *Journal of Agricultural and Food Chemistry, 52(18)*, 5670-5676, https://doi.org/10.1021/jf049512d

22. Tu, N.T.M., Onishi, Y., Choi, H.-S., Kondo, Y., Bassore, S.M., Ukeda, H., Sawamura, M. (2002) Characteristic odor components of Citrus sphaerocarpa Tanaka (Kabosu) cold-pressed peel oil. *Journal of Agricultural and Food Chemistry, 50(10)*, 2908-2913, https://doi.org/10.1021/jf011578a
