# Supplementary material for: Leaf volatile and nonvolatile metabolites show different levels of specificity in response to herbivory
Source: Ecol Evol. 2023 May 29;13(5):e10123. doi: 10.1002/ece3.10123 (PMC10225982; doi:10.1002/ece3.10123)
Supplement: Supplementary file 3 — Appendix S3 [file ECE3-13-e10123-s001.docx]

**Leaf volatile and non-volatile metabolites show different levels of specificity in response to herbivory**

**Supplementary information – Appendix S3**

**
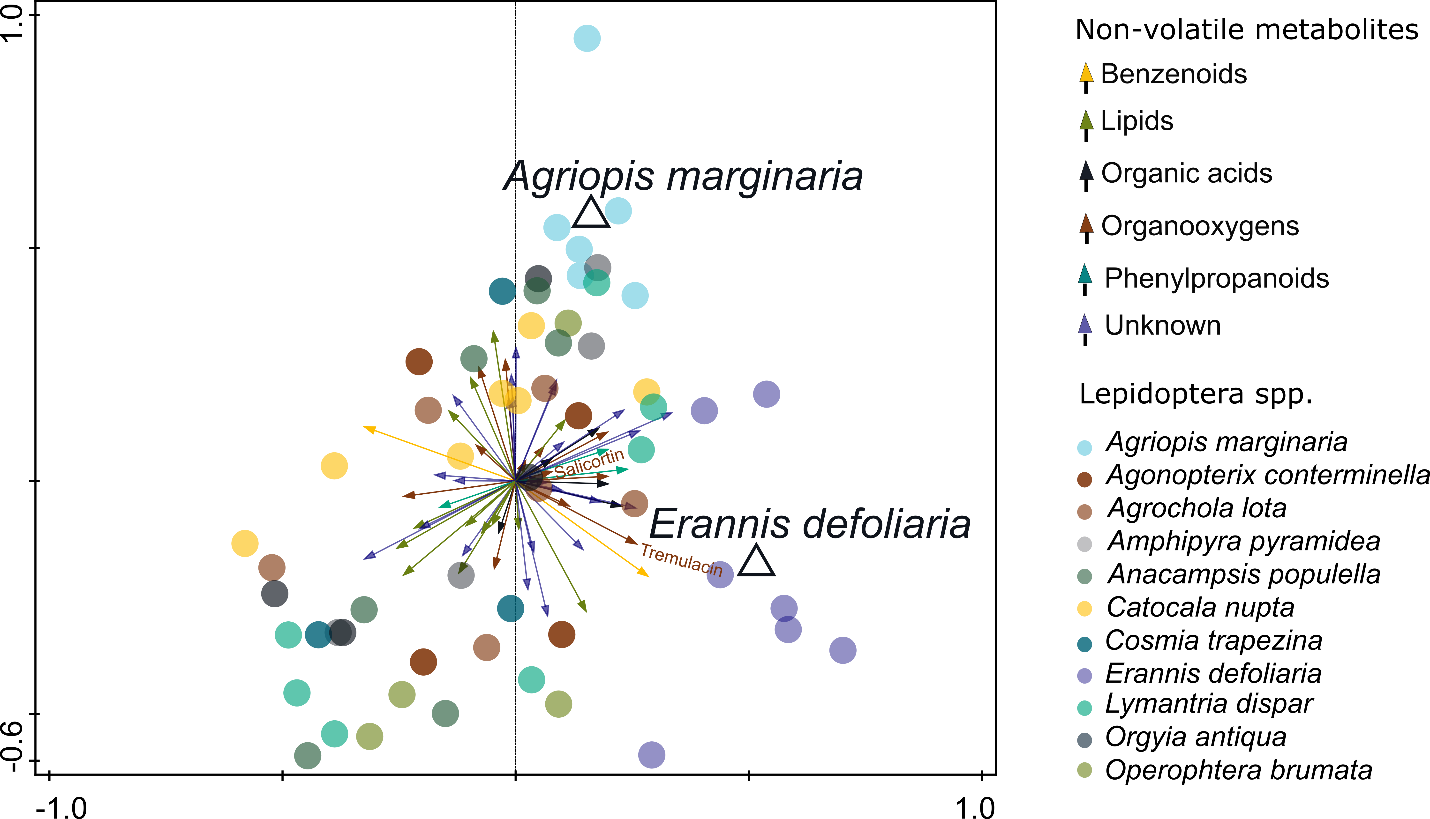
**

**Figure S2**. The correlation between non-volatile metabolites and individual species of Lepidoptera as analyzed with an RDA with forward selection. The species that best correlated to the variation in leaf metabolites included the two generalists *Erannis defoliaria* (pseudo-F=1.6, p=0.0552) and *Agriopsis marginaria* (pseudo-F=1.6, p=0.0708). The selected species, however, had only a marginally significant effect on the variation in leaf metabolites and jointly explained 2.04% of the adjusted variation in leaf metabolites. Individual non-volatile leaf metabolites are shown as coloured arrows, plant replicates are represented by the circles that are colour-coded according to herbivore treatments and controls. The empty triangles represent the centroids (mean point position) of each herbivore species, which represents the scores of samples belonging to that class (factor level).

**
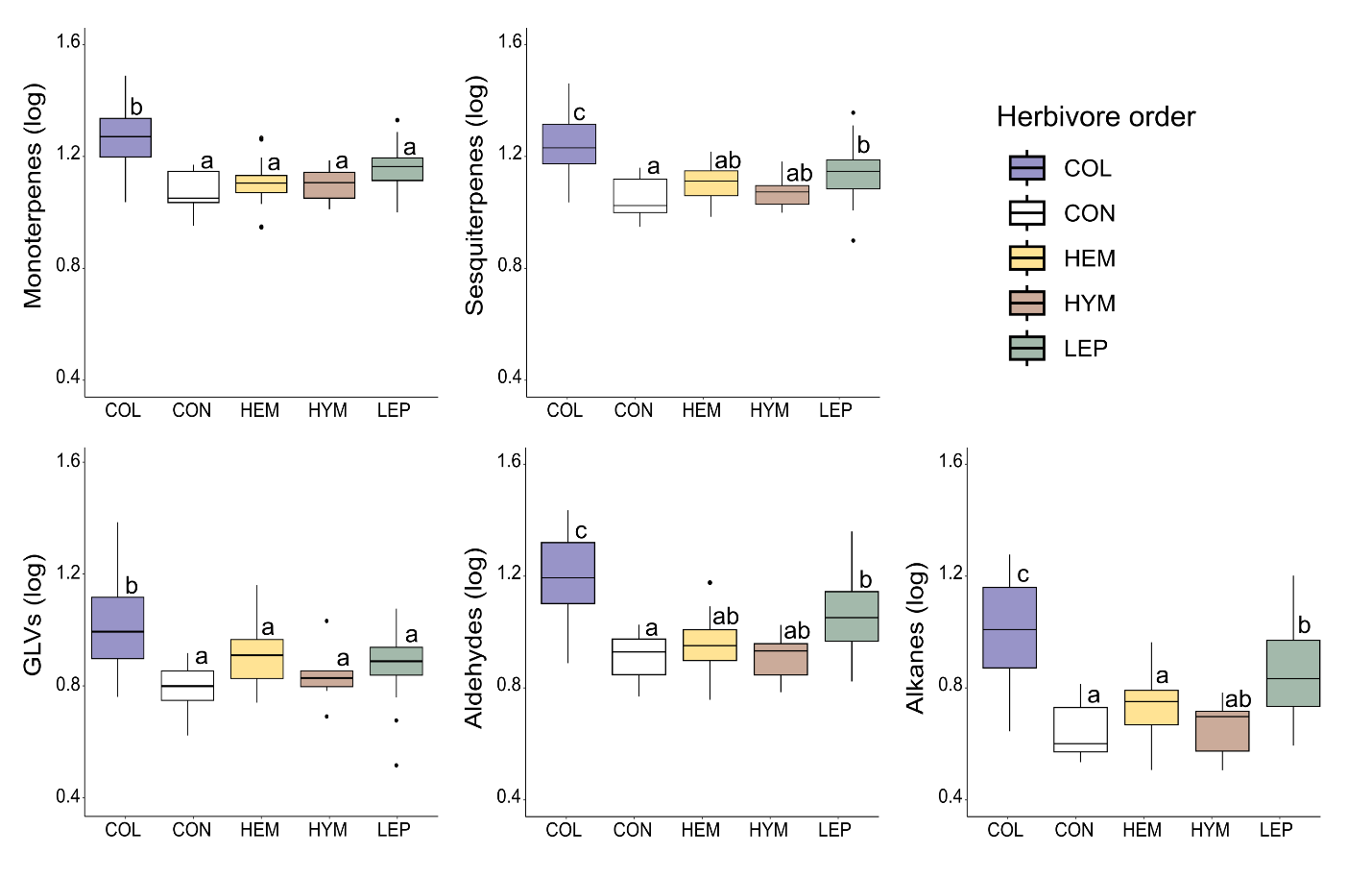
Figure S3.** Differences in the average emissions (peak under area) of different classes of VOCs among herbivore orders and control samples. The boxes show the first to the third quartile with the medians as horizontal lines, the whiskers show range. Significant differences between treatments (colour coded) as indicated by post-hoc Tukey tests are marked with letters above the boxes.

**
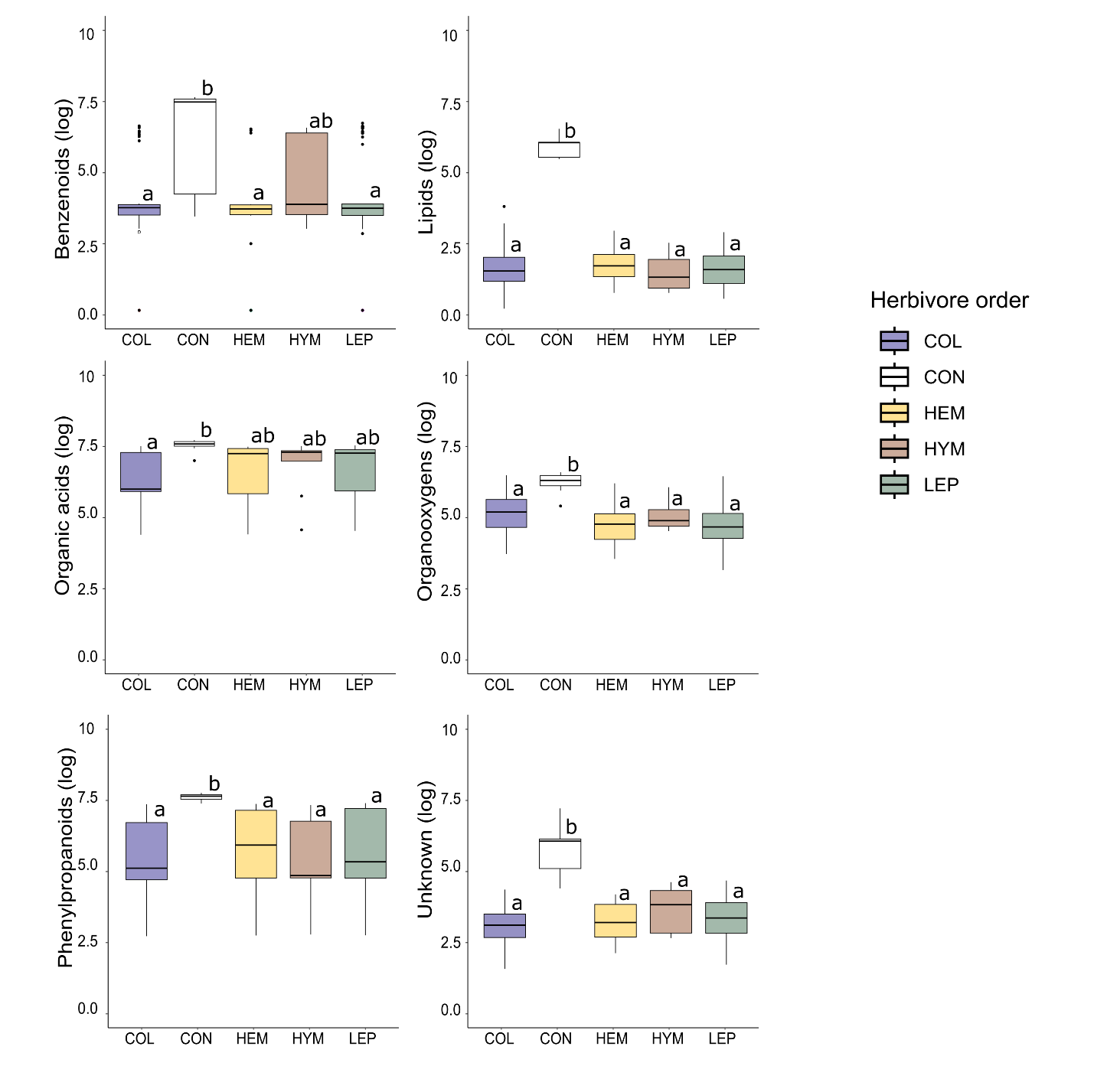
**

**Figure S4.** Differences in the average concentration (area under the peaks/mg) of different classes of non-volatile metabolites among herbivore orders and control samples. The boxes show the first to the third quartile with the medians as horizontal lines, the whiskers show range. Significant differences between treatments (colour coded) as indicated by post-hoc Tukey tests are marked with letters above the boxes.
